# Computer-aided detection of tuberculosis from chest radiographs in a tuberculosis prevalence survey in South Africa: external validation and modelled impacts of commercially available artificial intelligence software

**DOI:** 10.1016/S2589-7500(24)00118-3

**Published:** 2024-07-19

**Authors:** Zhi Zhen Qin, Martie Van der Walt, Sizulu Moyo, Farzana Ismail, Phaleng Maribe, Claudia M Denkinger, Sarah Zaidi, Rachael Barrett, Lindiwe Mvusi, Nkateko Mkhondo, Khangelani Zuma, Samuel Manda, Lisa Koeppel, Thuli Mthiyane, Jacob Creswell

**Affiliations:** a Stop TB Partnership, Geneva, Switzerland; b Department of Infectious Disease and Tropical Medicine, Heidelberg University Hospital, German Center for Infection Research (partner site), Heidelberg, Germany; c South Africa Medical Research Council, Pretoria, South Africa; d Human Sciences Research Council, Human and Social Capabilities Division, Cape Town, South Africa; e National Institute for Communicable Diseases, Pretoria, South Africa; f South African National Department of Health, Cape Town, South Africa; g World Health Organization, Pretoria, South Africa

## Abstract

**Background:**

Computer-aided detection (CAD) can help identify people with active tuberculosis left undetected. However, few studies have compared the performance of commercially available CAD products for screening in high tuberculosis and high HIV settings, and there is poor understanding of threshold selection across products in different populations. We aimed to compare CAD products' performance, with further analyses on subgroup performance and threshold selection.

**Methods:**

We evaluated 12 CAD products on a case–control sample of participants from a South African tuberculosis prevalence survey. Only those with microbiological test results were eligible. The primary outcome was comparing products' accuracy using the area under the receiver operating characteristic curve (AUC) against microbiological evidence. Threshold analyses were performed based on pre-defined criteria and across all thresholds. We conducted subgroup analyses including age, gender, HIV status, previous tuberculosis history, symptoms presence, and current smoking status.

**Findings:**

Of the 774 people included, 516 were bacteriologically negative and 258 were bacteriologically positive. Diverse accuracy was noted: Lunit and Nexus had AUCs near 0·9, followed by qXR, JF CXR-2, InferRead, Xvision, and ChestEye (AUCs 0·8–0·9). XrayAME, RADIFY, and TiSepX-TB had AUC under 0·8. Thresholds varied notably across these products and different versions of the same products. Certain products (Lunit, Nexus, JF CXR-2, and qXR) maintained high sensitivity (>90%) across a wide threshold range while reducing the number of individuals requiring confirmatory diagnostic testing. All products generally performed worst in older individuals, people with previous tuberculosis, and people with HIV. Variations in thresholds, sensitivity, and specificity existed across groups and settings.

**Interpretation:**

Several previously unevaluated products performed similarly to those evaluated by WHO. Thresholds differed across products and demographic subgroups. The rapid emergence of products and versions necessitates a global strategy to validate new versions and software to support CAD product and threshold selections.

**Funding:**

Government of Canada.

## Introduction

Tuberculosis is a curable disease, yet in 2022 it remained the world's second leading cause of death from a single infectious agent.^1^ A major contributor to high tuberculosis mortality rates is underdiagnosis. In 2022, 3·1 million of the estimated 10·6 million incident tuberculosis cases worldwide were not detected.^1^ Although chest x-ray is more sensitive than symptom screening, its effectiveness for tuberculosis screening has been reduced by high inter-reader and intrareader variability, modest reproducibility among human readers, and a short supply of radiologists in high burden countries.^2^, ^3^, ^4^, ^5^ To meet the global goal of ending tuberculosis by 2030 evidence-based use of innovative screening tools is required.^6^

Artificial neural networks are artificial intelligence (AI) designed to mimic human cognition to recognise patterns.^7^, ^8^ One powerful application of this is medical image interpretation.^7^ Computer-aided detection (CAD) products use AI to analyse chest x-ray images and detect tuberculosis-associated abnormalities. CAD products have been recommended by WHO for interpreting chest x-rays to triage and screen for tuberculosis in people aged 15 years and older instead of human readers.^9^ However, this recommendation does not stipulate specific products, and suggests more research is needed on performance in sub-populations. Meanwhile, the market for CAD solutions continues to grow, with 16 products on the market and more in development as of May, 2024.^10^, ^11^ Evidence on specific versions of certain products, and other products and software updates await independent scrutiny. A 2021 evaluation compared 12 products;^12^ however, this was conducted in a predominantly elderly population, with 72% of the total sample being drawn from individuals aged 55 years or older, and less than 1% having HIV, leaving a paucity of evidence on performance in a screening context, the general population, and high HIV-prevalence populations. HIV is relevant to tuberculosis screening as it can affect the presentation of abnormalities on chest x-rays.

**Research in context**

**Evidence before this study**

We searched PubMed for relevant literature from January, 2012 to May, 2024 in English, using the following search terms: ((Tuberculosis[MeSH]) OR (“tuberculosis”[tiab])) AND ((“artificial intelligence”[tiab]) OR (“computer-aided interpretation”[tiab]) OR (“computer aided detection”[tiab]) OR (“deep learning”[tiab]) OR (“convolutional neural networks”[tiab]) OR (“machine learning”[tiab]) OR (“automatic”[tiab]) OR (“computer aided reading”[tiab]) OR (“computer-aided reading”[tiab]) OR (“computer-aided detection”[tiab]) OR (“automated”[tiab])) OR ((“chest radiograph”[tiab) OR (“X-Ray” [tiab])). Of the 2987 results, we only included evaluation studies of the performance of commercially available computer-aided detection (CAD) software for detecting tuberculosis against a culture or Xpert bacteriological reference standard. Development studies, costing analyses, and evaluations against other reference standards (smear or radiological) were excluded, as were studies that evaluated CAD products' performance on children or medical images other than chest x-rays, as their findings are not comparable to this study. References from systematic reviews were consulted to identify additional studies. 18 studies were identified, with 15 of these evaluating the product CAD4TB on its own or with other software. There was some risk of bias as three of the studies included authors with a commercial interest in CAD4TB. Other frequently evaluated products were qXR and Lunit, with only two studies evaluating products other than these three. Nearly all studies measured accuracy using the area under the receiver operating characteristic curve (AUC), with analysis of the sensitivity and specificity at certain pre-defined thresholds or compared to human readers frequently included. The majority reported generally high accuracy in terms of AUC, although performance against human readers and at certain thresholds varied.

**Added value of this study**

This study adds to the existing literature by evaluating 12 CAD products, including some previously unevaluated, namely XVision, TiSepX-TB, and RADIFY. It is also to our knowledge the first evaluation of this many CAD products in a high HIV and high tuberculosis burden population, outlining how performance differs between subgroups. This study is also unique in including a threshold analysis section, showing how different programmatic criteria affect the choice of threshold score and how threshold scores vary both between products and sub-populations.

**Implications of all the available evidence**

This study shows that several CAD products perform well with high AUCs. On a practical implementation level, the study shows how thresholds can vary between sub-populations and for different programmatic targets, such as target sensitivity, target specificity, and confirmatory test referral rate. This might aid implementers in threshold selection in tuberculosis screening and triage programmes.

Most products generate a continuous abnormality score (a scale of 0–100 or 0–1) representing the likelihood of tuberculosis-related abnormalities in a chest x-ray. This score can be dichotomised at a threshold to give a binary outcome (suggestive of tuberculosis or not). Consequently, threshold selection directly influences the sensitivity and test-recall rate.^13^ There is an inherent trade-off in threshold selection. A lower threshold maximises sensitivity to detect true tuberculosis cases but will incur costs related to confirmatory diagnostic testing and reduced specificity. Conversely, a higher threshold will reduce the volume, and thus costs, of testing and likely identify only more severe cases, but reduce sensitivity.^13^ Currently, there is no standardised threshold across products in different populations; WHO advises against using one score for all contexts because CAD performance varies by population.^14^

We present a head-to-head comparison of 12 CAD products, which were commercially available between 2020 and 2023, in detecting bacteriologically confirmed tuberculosis. We aimed to analyse their performance in key sub-populations using data from the national tuberculosis prevalence survey in South Africa, a high tuberculosis and HIV-burden country. Furthermore, we aimed to identify the best thresholds for each product, depending on programmatic aims including target sensitivities of 90% and 80%, specificity of 70%, and a 10% chest x-ray abnormality rate within sub-populations.

## Methods

### Study design and procedures

**Figure 1:**
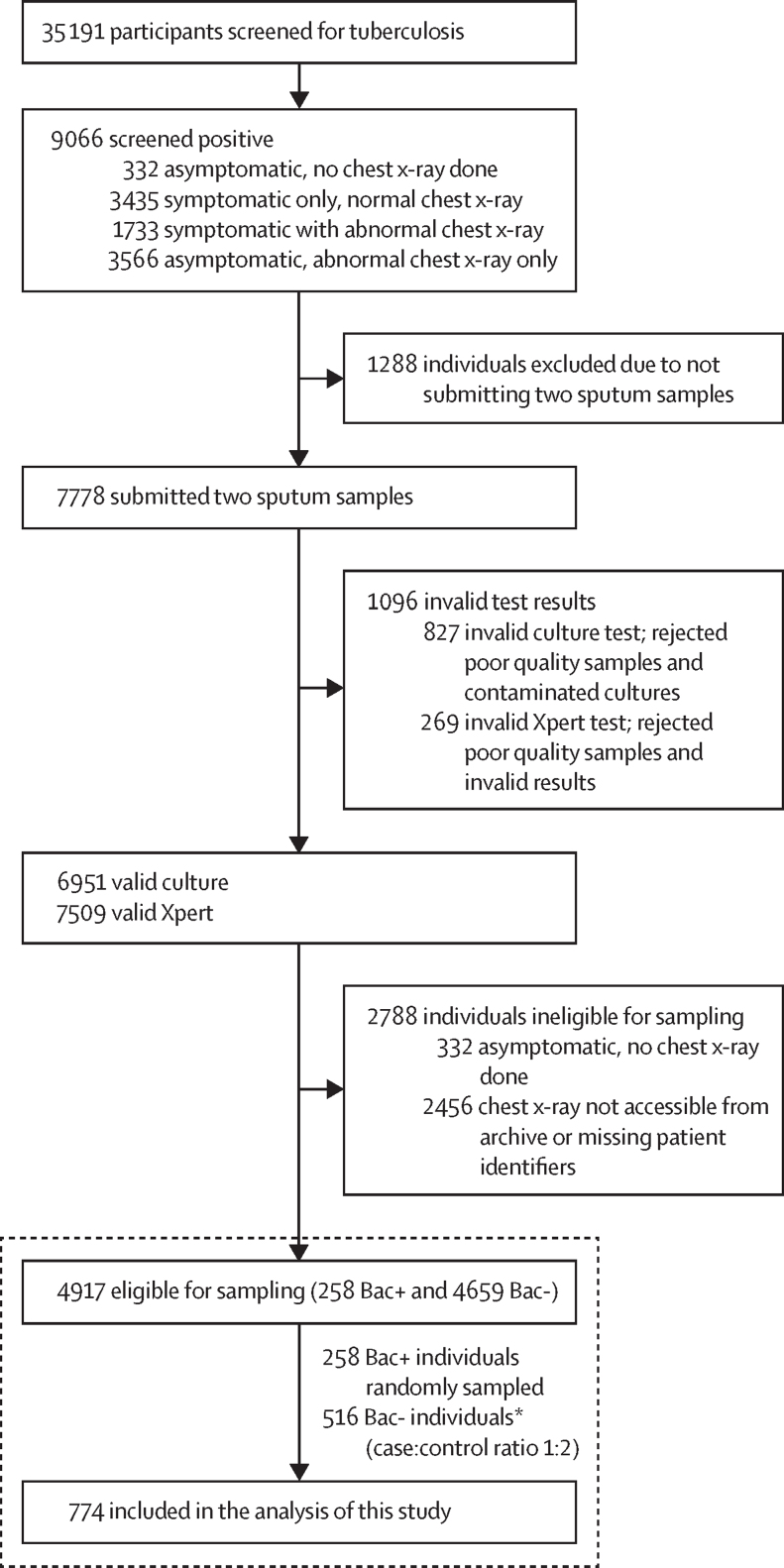
Overview of the data collection from the first national tuberculosis prevalence survey, South Africa Bac+=bacteriologically positive. Bac-=bacteriologically negative. *Bac+ and Bac- denote the bacteriological status determined by the composite microbiological reference standard in this study, where anyone with *Mycobacterium tuberculosis* detected by either Xpert Ultra or culture is referred to as Bac+.

This case–control evaluation used digital chest x-ray images and metadata from individuals 15 years or older who participated in the South African national tuberculosis prevalence survey between Aug 15, 2017 and July 28, 2019.^15^ During the survey, 35 191 individuals were screened using a WHO-recommended four-symptom-screen (comprising cough, fever, night sweats, or weight loss) and chest x-rays. Survey physicians (medical officers experienced in reading chest x-rays for tuberculosis) identified individuals with abnormal chest x-rays. 9066 individuals who reported symptoms or had an abnormal chest x-ray indicative of tuberculosis were asked to provide two sputum specimens.^15^ Sputum samples from 7778 individuals were processed using the GeneXpert MTB/RIF Ultra (Xpert; Cepheid, Sunnyvale, CA, USA) assay and MGIT liquid culture (culture; Bactec MGIT 960, Becton Dickinson, Franklin Lakes, NJ, USA). Of the individuals tested for tuberculosis, 6951 had valid culture results and 7509 had valid Xpert results.^15^ After excluding 332 asymptomatic individuals or those without chest x-rays, and 2456 individuals whose data were not accessible or missing patient identification, a total of 4917 participants were eligible for sampling (figure 1). Individuals without symptoms who had a clear chest x-ray and did not receive confirmatory testing were excluded from the sample; these might have included people with tuberculosis. In this study, we used Xpert and culture results to form a composite microbiology reference standard; cases were people considered bacteriologically positive if they tested positive for tuberculosis on either or both tests. Xpert trace results were only included as positive if culture was positive. Controls were those that did not have bacteriological confirmation of tuberculosis. Two other reference standards were used: bacteriologically positive by culture-only and bacteriologically positive by Xpert-only.

Between January, 2021 and August, 2021 we contacted the manufacturers of all eight commercially available CAD products (CAD4TB [Delft Imaging Systems, Netherlands], Genki [Deeptek, India], InferRead DR Chest [InferVision, China], Lunit INSIGHT CXR [Lunit, South Korea], qXR [Qure.ai, India], JF CXR-2 [JF Healthcare, China], XrayAME [Epcon, Belgium], and JVIEWER-X [JLK, South Korea]) for tuberculosis according to the latest 2021 landscape report,^10^ and five others (EnvisionIT [South Africa], MedicalIP [South Korea], Oxipit [Lithuania], RadiSen [South Korea], and VUNO [South Korea]) that were commercially available according to ai4hlth.org at the time of the study.^10^, ^11^ Finally, Nexus (Nexus CXR [GoogleResearch Model], South Africa) expressed interest to join this study in December, 2023 immediately following its market entry.

Participants' chest x-ray were stored on Stop TB Partnership's server using secure file transfer protocol. Each CAD software was installed to analyse the chest x-ray without any data transfer to the CAD developers. CAD readings were aggregated and exported. When dedicated cloud graphics processing capacity was needed, the same process was undertaken on a Google cloud server. All readings were performed independently and blind to clinical or demographic information. No training was performed, and developers had no access to images before the evaluation, except for four files used to test the process and check product compatibility which were excluded from sampling. Developers had no other involvement in the study.

### Statistical analysis

Assuming an expected sensitivity of 90%, and specificity of 70%, as per WHO target product profile of a triage test,^16^ with a precision level of 5%, the minimum required sample size for confirmed tuberculosis cases was 138, as per the CAD calibration toolkit.^14^, ^17^ However, to enhance statistical power, we included all 258 bacteriologically positive individuals from the primary prevalence survey and doubled this number by randomly selecting 516 bacteriologically negative individuals using a random number generator. The p value to compare the software was adjusted to 0·0042, using the Bonferroni correction to avoid inflating the type I error rate during multiple comparisons.

To compare overall product performance, we plotted the receiver operating characteristic curve for each product using the microbiology reference standard and culture-only and Xpert-only standards and calculated the area under the receiver operating characteristic curves (AUCs). A sensitivity analysis was performed to examine the AUC when using culture-only and Xpert-only standards. The demographic characteristics and distribution of AI scores for each product were compared by bacteriological status using the Chi-squared test for categorical variables and a two-sample *t*-test for continuous variables.

Each product's performance was then evaluated against WHO's target product profile sensitivity of 90% and specificity of 70%.^18^ Because sensitivity and specificity are in a constant trade-off, and change depending on the threshold, we first calculated the sensitivity and specificity of all products across all thresholds between 0 and 1 using the composite microbiology reference standard. We found the threshold closest to 90% sensitivity and checked its specificity against the 70% target. Then, we found the threshold for 70% specificity and compared its sensitivity with the 90% target.

We modelled the effect of threshold selection in a hypothetical population of 9000 people with a 2% tuberculosis prevalence akin to that in the original survey.^15^ This involved applying calculated sensitivities and specificities to determine x-ray abnormality and confirmation test rates, assuming only the individuals with a CAD score above the threshold would be subject to confirmation tests. Criteria based on common programmatic targets, including sensitivity, specificity, and test referral rate were defined to evaluate threshold selection. We also analysed the dynamics between sensitivity, abnormality score, abnormal chest x-ray rate, and confirmation test positive rate across all thresholds between 0 and 1. Details of the model's calculations are in the appendix (p 2). Importantly, since the modelled data were based on the prevalence of people either with symptoms or an abnormal chest x-ray, the results might not apply to the general population.

For threshold analysis, we first set the threshold selection criteria to achieve 90% and 80% sensitivity, 70% specificity, and 10% x-ray abnormality rate, in addition to imposing the mid-point (0·5) as the threshold, as this is recommended by some manufacturers. We reported the corresponding thresholds, sensitivity, and specificity based on these criteria. For the second phase, we plotted sensitivity over a continuous range of thresholds, abnormal chest x-ray rate over a continuous range of thresholds, the trade-off between sensitivity and proportion of confirmation tests saved, and the confirmation test positive rate over a continuous range of thresholds of each product.

To investigate if performance differs in sub-populations, we stratified the study population by gender, age, presence of tuberculosis symptoms, previous tuberculosis history, current smoking status, and HIV status (appendix p 2).^15^ We calculated each product's AUC and identified optimal thresholds for achieving desired sensitivity and specificity levels in these groups. Multivariable analysis was conducted to investigate how combining demographic and clinical variables with CAD scores affects performance (appendix pp 3–11). All calculations were conducted using R version 3.6.0.

A data-sharing request for anonymised chest x-ray was approved by the National Department of Health, South Africa, through the South African Medical Research Council (SAMRC). SAMRC provided the automatically anonymised and hashed chest x-ray images, ensuring no manual data handling occurred pre-anonymisation. Written informed consent was obtained from all participants, with additional assent and parental consent for those aged 15–18 years. Anonymised data were stored in Stop TB Partnership's secure file transfer protocol server accessible only to co-investigators. Ethical approval was obtained from SAMRC (EC002–3/2020) and Heidelberg (S-488/2021).

### Role of the funding source

The funder of the study was not involved in the study design, data collection, analysis, interpretation, or reporting.

## Results

We invited 13 companies to include their software: JLK, RadiSen, and VUNO declined to participate. The ten companies that consented were CAD4TB (version 7, Delft Imaging Systems, Netherlands), ChestEye (version 2.4, Oxipit.ai, Lithuania), Genki (version 20.12, DeepTek, India), InferRead DR Chest (version 1, Infervision, China), JF CXR-2 (version 2, JF Healthcare, China), Lunit INSIGHT CXR (version 4.9, Lunit, South Korea), qXR (version 3, Qure.ai, India), RADIFY (version 3.5.0c, Envisionit, South Africa), TiSepX-TB (version 1.0.0.0, MedicalIP, South Korea) and XrayAME (version 1, Epcon, Belgium). Two additional companies with commercially available products, Xvision (version 2.2.211, Mindfully Technologies, Romania) and Nexus CXR (version 1, Nexus, South Africa), contacted us during data collection and were included. Altogether, 12 products were evaluated (appendix p 12).

Of the 774 individuals included in this study (figure 1), 369 (47·7%) had an abnormal chest x-ray but no symptoms. Among the 258 (33·3%) of 774 bacteriologically positive individuals (cases), 189 (73·3%) were positive on both liquid culture and Xpert, 61 (23·6%) were positive on culture and not Xpert, and 70 (27·1%) were positive on Xpert and not culture. Cases were younger than bacteriologically negative individuals (controls), with a median age of 44·7 years (IQR 17·0) compared with 50·1 years (18·8); and had a greater proportion of individuals with a history of tuberculosis (35% [91/258] *vs* 21% [106/516]). Cases were more likely to have received tuberculosis treatment previously (31% [79/258]) than controls (20% [105/516). HIV infection rate also varied significantly between cases (25% [65/258]) and controls (15% [77/516]). Furthermore, bacteriologically positive individuals were less likely to report symptoms (43% [110/258]) compared to controls (57% [295/516]) or to smoke (48% [123/258] *vs* 60% [306/516]). As for chest x-ray findings by survey physicians, a smaller proportion of bacteriologically positive chest x-rays were labelled “normal” (7% [18/258] *vs* 37% [191/516]), and a greater proportion were graded “abnormal suggestive of tuberculosis” (93% [240/258] *vs* 61% [317/516]). All products allocated higher median scores to the cases compared with the controls (table 1). The appendix (p 13) shows the histograms CAD scores by bacteriologically confirmed status and tuberculosis history.

**Table 1:** Baseline characteristics

		**Overall (N=774)**	Bacteriologically positive*(n=258)	Bacteriologically negative*(n=516)	**p value**
Gender		..	..	..	0·25
	Male	396 (51%)	140 (54%)	256 (50%)	..
	Female	378 (49%)	118 (46%)	260 (50%)	..
Age		48·3 (18·3)	44·7 (17·0)	50·1 (18·8)	<0·01
Age group		..	..	..	<0·01
	15 to <35 years	219 (28%)	84 (33%)	135 (26%)	..
	35 to <55 years	249 (32%)	103 (40%)	146 (28%)	..
	≥55 years	306 (40%)	71 (28%)	235 (46%)	..
Previous history of tuberculosis (yes)		197 (26%)	91 (35%)	106 (21%)	<0·01
	Currently receiving treatment for tuberculosis	28 (4%)	23 (9%)	5 (1%)	<0·01
	Previously received treatment for tuberculosis	184 (24%)	79 (31%)	105 (20%)	<0·01
HIV status					
	HIV+	142 (18%)	65 (25%)	77 (15%)	<0·01
	HIV–	488 (63%)	150 (58%)	338 (66%)	..
	Unknown	144 (19%)	43 (17%)	101 (20%)	..
Any symptoms (yes)		405 (52%)	110 (43%)	295 (57%)	<0·01
	Cough >2 weeks	148 (19%)	49 (19%)	99 (19%)	1
	Cough	258 (33%)	77 (30%)	181 (35%)	0·16
	Fever	113 (15%)	31 (12%)	82 (16%)	0·17
	Night sweats	178 (23%)	52 (20%)	126 (25%)	0·20
	Weight loss	141 (18%)	46 (18%)	95 (18%)	0·91
Physician chest x-ray findings		..	..	..	<0·01
	Normal	209 (27%)	18 (7%)	191 (37%)	..
	Abnormal—suggestive of tuberculosis	557 (72%)	240 (93%)	317 (61%)	..
	Abnormal—other	8 (1%)	0	8 (2%)	..
	Poor image quality	16 (2%)	4 (2%)	12 (2%)	..
	Abnormal chest x-ray but no symptoms	369 (48%)	148 (57%)	221 (43%)	0·29
Current smoking status					<0·01
	Yes	429 (56%)	123 (48%)	306 (60%)	..
	No	345 (44%)	135 (52%)	210 (40%)	..
Diabetes (any type)		..	..	..	0·08
	Yes	58 (8%)	12 (5%)	46 (9%)	..
	No	704 (91%)	243 (94%)	461 (89%)	..
	Unknown	12 (2%)	3 (1%)	9 (2%)	..
Culture result		..	..	..	<0·01
	Positive for *M tuberculosis*	189 (24%)	189 (73%)	0	..
	Negative for *M tuberculosis*	516 (67%)	57 (22%)	459 (89%)	..
	Contaminated	29 (4%)	8 (3%)	21 (4%)	..
	Non-tuberculous mycobacteria	13 (2%)	0	13 (3%)	..
	Not done	27 (4%)	4 (2%)	23 (5%)	..
Xpert result		..	..	..	<0·01
	Positive	197 (26%)	197 (76%)	0	..
	Trace positive	26 (3%)	21 (8%)	5 (1%)	..
	Negative	539 (70%)	38 (15%)	501 (97%)	..
	Invalid	3 (0·4%)	0	3 (1%)	..
	Not done	9 (1%)	2 (1%)	7 (1%)	..
CAD reading					
	JF CXR-2	0·38 (0·03–0·93)	0·95 (0·82–0·98)	0·08 (0·01–0·62)	<0·01
	Lunit	0·09 (0·01–0·82)	0·90 (0·70–0·96)	0·02 (0·01–0·14)	<0·01
	Nexus	0·52 (0·15–0·84)	0·89 (0·78–0·93)	0·25 (0·10–0·61)	<0·01
	CAD4TB	8 (1–59)	70 (27–91)	2 (1–18)	<0·01
	qXR	0·30 (0·05–0·88)	0·92 (0·66–0·97)	0·09 (0·03–0·43)	<0·01
	InferRead	0·35 (0·17–0·71)	0·74 (0·57–0·84)	0·24 (0·14–0·41)	<0·01
	Genki	0·07 (0·00–0·57)	0·62 (0·35–0·84)	0·01 (0·00–0·17)	<0·01
	XrayAME	0·08 (0·02–0·44)	0·45 (0·08–0·90)	0·04 (0·01–0·17)	<0·01
	ChestEye	0·10 (0·04–0·42)	0·46 (0·19–0·62)	0·06 (0·03–0·14)	<0·01
	Xvision	0·14 (0·08–0·46)	0·51 (0·27–0·62)	0·10 (0·07–0·16)	<0·01
	TiSepX-TB	0·26 (0·14–0·51)	0·60 (0·32–0·88)	0·19 (0·13–0·31)	<0·01
	RADIFY	0·47 (0·00–0·60)	0·54 (0·23–0·84)	0·43 (0·00–0·59)	<0·01

Data are n (%) or median (IQR). CAD=computer-aided detection.

* Bacteriologically positive and bacteriologically negative denote the bacteriological status determined by the composite microbiological reference standard in this study, where anyone with *Mycobacterium tuberculosis* detected by either Xpert Ultra or culture is referred to as bacteriologically positive.

**Figure 2:**
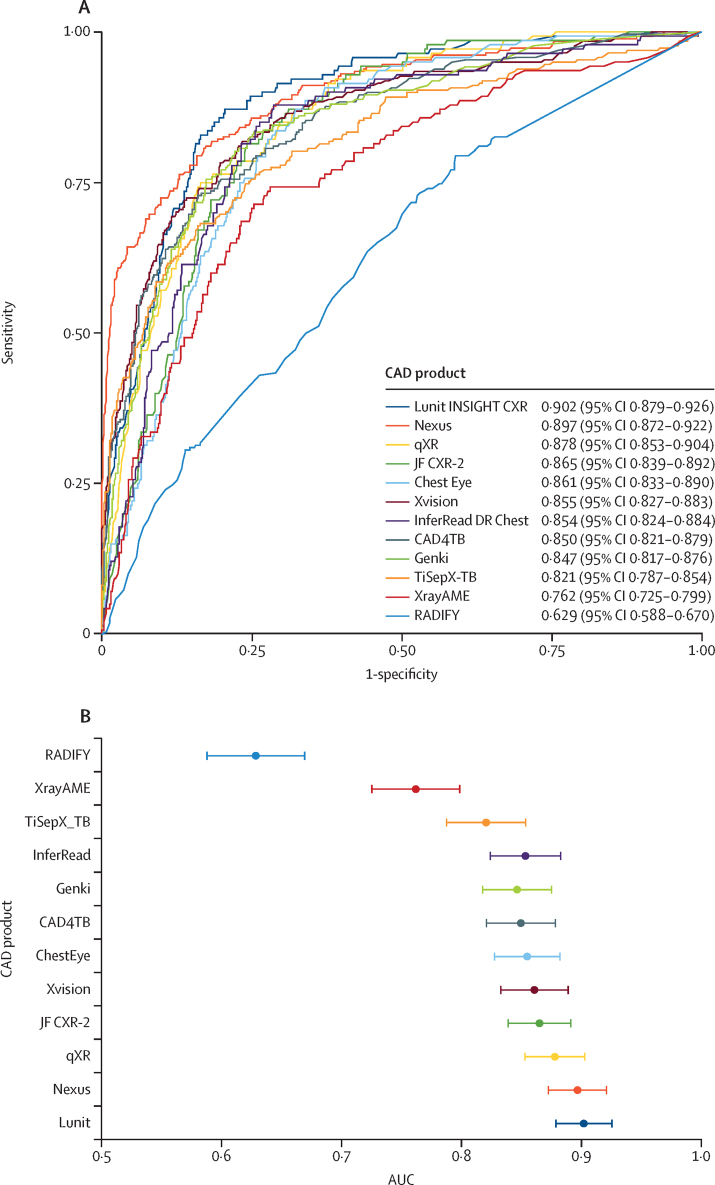
The receiver operating characteristic curves against the composite microbiology reference standard (A) and the AUCs (B) of 12 commercially available CAD products AUC=area under the receiver operating characteristic curve. CAD=computer-aided detection.

The receiver operating characteristic and overall performance (AUC) for each product against the composite microbiology reference standard are shown in figure 2 and appendix p 14. Five products had AUCs higher than 0·86: Lunit (0·902 [95% CI 0·879–0·926]), Nexus (0·897 [0·872–0·922]), qXR (0·878 [0·853–0·904]), JF CXR-2 (0·865 [0·839–0·892]), and Xvision (0·861 [0·833–0·890]). Of these, Lunit and Nexus statistically significantly outperformed all other products (p<0·0042), except Nexus did not outperform qXR, and the others had comparable AUCs (appendix p 15). Four further products had AUCs between 0·84 and 0·86 and performed statistically similarly to each other: ChestEye, InferRead DR Chest, CAD4TB, and Genki. Three products had lower performance: TiSepX-TB, XrayAME, and RADIFY, with the latter two having statistically significantly lower AUCs than all other products (p<0·0042). Sensitivity analysis results using alternative standards are in the appendix (p 14).

At 90% sensitivity, five products achieved greater than 60% specificity: Lunit, Nexus, JF CXR-2, qXR, and ChestEye, with Lunit and Nexus having CIs overlapping the target product profile target of 70% specificity (table 2). Xvision, CAD4TB, InferRead, and Genki achieved specificity between 50% and 60%, with performance close to that of JF CXR-2, qXR, and ChestEye with some overlap in CIs. TiSepX-TB, XrayAME, and RADIFY had specificities less than 50%, with RADIFY statistically significantly less specific than all other products (appendix p 15).

**Table 2:** Computer-aided detection performance compared with 90% sensitivity and 70% specificity target values

	**Threshold**	**Sensitivity**	**Specificity**
**Threshold to match 90% sensitivity**			
Lunit	0·07	89·9% (85·6–93·3)	67·7% (63·5–71·7)
Nexus	0·48	89·9% (85·6–93·3)	67·1% (62·9–71·2)
JF CXR-2	0·23	89·5% (85·1–93·0)	62·7% (58·3–66·9)
qXR	0·18	90·3% (86·0–93·6)	62·3% (57·9–66·5)
ChestEye	0·08	89·1% (84·7–92·7)	61·3% (57·0–65·5)
Xvision	0·11	89·9% (85·6–93·3)	58·6% (54·2–62·9)
CAD4TB	3	89·9% (85·6–93·3)	55·7% (51·3–60·0)
InferRead	0·26	90·3% (86·0–93·6)	54·9% (50·5–59·3)
Genki	0·02	89·5% (85·1–93·0)	54·5% (50·1–58·9)
TiSepX-TB	0·18	89·9% (85·6–93·3)	48·0% (43·6–52·4)
XrayAME	0·02	88·4% (83·8–92·0)*	36·9% (32·8–41·3)
RADIFY	0·02	82·6% (77·4–87·0)*	32·5% (28·5–36·7)
**Threshold to match 70% specificity**			
Lunit	0·09	89·5% (85·1–93·0)	70·2% (66·1–74·1)
Nexus	0·54	88·8% (84·3–92·3)	69·8% (65·7–73·8)
qXR	0·32	86·8% (82·1–90·7)	70·2% (66·1–74·1)
JF CXR-2	0·4	86·4% (81·6–90·4)	70·0% (65·9–73·9)
ChestEye	0·11	86·0% (81·2–90·0)	69·6% (65·5–73·6)
InferRead	0·37	85·7% (80·8–89·7)	70·4% (66·3–74·3)
Xvision	0·14	85·7% (80·8–89·7)	69·6% (65·5–73·6)
Genki	0·09	84·5% (79·5–88·7)	70·6% (66·5–74·5)
CAD4TB	12	81·0% (75·7–85·6)	70·6% (66·5–74·5)
TiSepX-TB	0·29	77·5% (71·9–82·5)	70·8% (66·7–74·7)
XrayAME	0·13	68·6% (62·6–74·2)	70·6% (66·5–74·5)
RADIFY	0·57	43·4% (37·3–49·7)	71·0% (66·9–74·9)†

* The closest sensitivity to 90%.

† The closest specificity to 70%.

At 70% specificity, five products (Lunit, Nexus, qXR, JF CXR-2, and ChestEye) performed similarly to the target product profile with sensitivities greater than 86% and CIs overlapping the 90% target (table 2). Several other CAD products also achieved a similar performance with sensitivity point estimates between 81% and 85% (InferRead, Xvision, Genki, and CAD4TB); CIs did not reach the target value. Meanwhile, TiSepX-TB, XrayAME, and RADIFY had sensitivities less than 80%, with XrayAME and RADIFY having statistically significantly lower sensitivities than all other products except TiSepX-TB.

For the same criterion, there were differences in threshold scores across different CAD products. For example, to achieve 80% sensitivity, the threshold was 0·75 for JF CXR-2 and 0·05 for XrayAME. At 60% specificity, Lunit required a threshold of 0·04, compared with 0·5 for RADIFY (appendix pp 16–19).

In the scenario where confirmation tests were available for only 10% of presumptive tuberculosis cases, the associated thresholds ranged from 0·4 to 0·9. Lunit and Nexus performed best, with sensitivities of 75·2% (95% CI 69·5–80·3%) and 70·5% (64·6–76·0%).

At a fixed threshold of 0·5, sensitivity and specificity of different CAD products varied. The sensitivity of Nexus was highest at 89·1% (95% CI 84·7–92·7%), followed by JF CXR-2 (86·0% [81·2–90·0%]), qXR (80·6% [75·3–85·3%]), and Lunit (80·2% [74·8–84·9%]). In comparison, ChestEye and XrayAME were below 50% sensitivity. This variation underscores the non-transferability of thresholds between products.

Increasing thresholds generally reduced sensitivity and abnormal chest x-ray rates. Lunit, Nexus, JF CXR-2, and qXR maintained high sensitivity over a wider range of thresholds, resulting in more individuals being triaged by x-ray. For these CADs, as the threshold increased from 0 to 0·7–0·8, sensitivity initially stayed above 90% and remained over 70% until it reached that range, while the proportion of individuals classified as having abnormal chest x-ray reduced from 100% to below 30%. CAD4TB, Genki, XrayAME, RADIFY, Xvision, and ChestEye displayed an almost linear relationship across abnormality scores, and required a very low threshold to maintain sensitivity near 90% (appendix pp 19–20).

Several products performed worse in individuals with a history of tuberculosis than in those without (p <0·0042, appendix pp 20–24). All products except RADIFY performed better for younger individuals (aged 15–34 years) than individuals aged 55 years or older. Only qXR performed statistically significantly better in individuals aged 35–54 years than individuals aged 55 years or older. There were no statistically significant differences between people aged 15–34 years and people aged 35–54 years. CAD products generally had lower AUCs in people living with HIV, but none were statistically significant, similar to variations based on symptoms, gender, and smoking status (appendix pp 20–24).

Each product was assessed at the threshold of 0·5 (appendix pp 25–34). qXR's sensitivity was higher in individuals with a history of past tuberculosis (86·8% [95% CI 78·1–93·0%]) than in individuals without a history of tuberculosis (77·2% [70·1–83·4%]), but its specificity was lower in former tuberculosis patients (37·3% [28·5–47·7%] *vs* 87·3% [83·7–90·4%]). Differences in sensitivity and specificity were not statistically significant between people living with HIV and HIV-negative individuals. Sensitivity was not statistically significantly different between symptomatic and asymptomatic individuals; specificity was statistically significantly higher in symptomatic people. Sensitivity was statistically significantly higher in people aged 15–34 years and people aged 35–54 years than in people older than 55 years. Specificity was statistically significantly higher in people aged 15–34 years than in people aged 35–54 years and people older than 55 years.

Threshold adjustments helped meet programmatic targets in different sub-populations (appendix pp 35–44). qXR required a threshold of 0·14 to achieve 90% sensitivity in new cases versus 0·34 in people with a history of tuberculosis, but its specificity dropped in people with a history of tuberculosis (33·0% [95% CI 24·2–42·8%]) compared with those without a history of tuberculosis (66·2% [61·4–70·7%]).

## Discussion

This study is the first to our knowledge to independently validate a comprehensive list of commercial AI products for tuberculosis in a high tuberculosis and HIV prevalence screening setting. The study shows that products not included in WHO guidelines (XVision, Nexus, ChestEye, JF CXR-2, InferRead, and Genki) performed similarly to WHO-reviewed ones.^9^ The rapid pace of software development, and improvement, requires flexibility from regulators and underscores the need for an impartial evaluation centre able to update and publish results. The evidence-based implementation of CAD products would benefit from the establishment of a coordinated, global evaluation effort.

With the WHO tuberculosis screening guidelines incorporating the use of CAD products, the question of product selection is increasingly important, as well as threshold selection in different settings and for different populations. Our results highlight the marked difference in required thresholds for the same criterion from different products. This implies that if a country deploys various products, the use of the same threshold will result in different sensitivity, specificity, and chest x-ray abnormal rate. Under the mid-point threshold, the sensitivity of all products fell short of the 90% sensitivity target product profile target, although Nexus performed statistically similarly to the targets. Furthermore, several others achieved sensitivities from 70% to 86%. This similarity in performance between most products elevates the importance of additional considerations such as service level, user interface, pricing, and additional product features (for example, ability to detect non-tuberculosis abnormalities) when selecting CAD products for programmes.

Despite their different neural networks, the products evaluated show similar subgroup bias, universally performing worse in older age groups, except RADIFY, and some also performed worse in those with a history of tuberculosis, corroborating previous literature.^12^, ^18^ These observations might be related because older age groups in high-burden regions are more likely to have previously had tuberculosis, or other scarring on the lungs, which reduces the accuracy of both CAD and human readers. With more people surviving tuberculosis and living longer, this is likely to become a bigger challenge. HIV is a key risk factor for tuberculosis and our study is the first to our knowledge to independently evaluate the performance of multiple products depending on HIV status. HIV infection is hypothesised to affect the presentation of tuberculosis on chest x-ray, which has been shown to impact human reader interpretation.^19^, ^20^ Similarly, our results showed all CAD products perform worse in people living with HIV in terms of AUC, but the difference was not statistically significant. In contrast, earlier evaluations of CAD4TB demonstrated significantly worse performance in people with HIV,^21^, ^22^ although this analysis and another recent evaluation^23^ in South Africa demonstrated no significant variation, which could imply algorithm improvement.

The abnormality score required in this study to achieve 90% sensitivity when using CAD4TB differs greatly from a previous evaluation using the same product version which cites a threshold of 50 for CAD4TB.^18^ Using 50 in this study's population would result in sensitivity of only 64·0%. Similarly, the thresholds required to reach 90% sensitivity for qXR, InferRead, Lunit, and JF chest x-ray are all different between the two studies.^18^ In another evaluation using the same software versions for qXR, CAD4TB, and InferRead as this study, the thresholds to achieve sensitivity greater than 95% were 0·441 (qXR), 46 (CAD4TB), and 0·538 (InferRead).^12^ Using the same thresholds in this study would result in lower respective sensitivities of 83%, 66%, and 76%. Implementers such as national tuberculosis programmes seeking to use CAD should therefore be wary of extrapolating thresholds quoted in literature. On-site operational research is crucial to select the optimal threshold.

Our presentation of varied performance refutes the notion that a universally recommended threshold could be appropriate at present. Instead, it highlights the need for implementers to select tailored thresholds depending on the use case and characteristics of the population screened. This study therefore provides an example of how to choose thresholds for distinct groups served by an intervention. Further guidance on carrying out operational research to choose the most appropriate threshold can be found in the CAD calibration toolkit developed by WHO and the Special Programme for Research and Training in Tropical Diseases.^14^

Our study has several limitations. Firstly, during prevalence survey data collection, individuals were relied on to self-report demographic, lifestyle, and clinical data. Any inaccuracy when self-reporting could be carried forward to our sub-analyses. In the survey, many individuals had not provided HIV co-infection data or did not know their status for some health data; hence, we had limited statistical power to investigate some comorbidities like silicosis, which would have been significant given the high tuberculosis prevalence among miners. A low number of people were receiving treatment for tuberculosis at the time of the prevalence survey, and we have not evaluated CAD here as a separate subgroup due to low power. Instead, these were aggregated with those with a history of tuberculosis. Moreover, ethnicity data were not collected (it was not in the protocol for this study, although data on race were included in the prevalence survey and the majority of participants were Black South Africans), so we were unable to compare performance between ethnicities. Furthermore, among participants without symptoms nor abnormal x-ray assessed by the survey physicians there could be bacteriologically confirmed tuberculosis, and these participants were not included in the analysis. This means our reported sample estimates of sensitivity and specificity are likely higher than true population estimates, which limits generalisability. Importantly, children below the age of 15 years were not eligible for participation in the prevalence survey, and we caution against extrapolating the results of our evaluation to a population younger than 15 years as none of the products were licensed for use in a younger age group. Further research should be performed to assess CAD accuracy in child and adolescent populations, especially given the high childhood tuberculosis burden.^1^

There are limitations with our sample size calculations. As we were comparing 12 products, our study is underpowered to detect differences in performance between the software. Using the Bonferroni correction, a sample size of 291 diseased cases would be required to achieve a target sensitivity of 90%. However, we only obtained 258 positive tuberculosis cases. This means the lack of difference between the products could be due to a small sample size; we urge caution in making strong claims based on this. Additionally, in the subgroup analyses, the number of disease cases in each group might be lower, which could further limit statistical power.

In conclusion, many CAD products performed at a similarly high level in a high HIV and tuberculosis-burden population, but there are differences in algorithms. No significant difference was noted in people living with HIV compared with HIV-naive populations in product AUCs, thus further evaluations in this and other populations are necessary. Several context-specific factors should be considered when deciding on which product and threshold to use, including population screened, diagnostic test availability, and comorbidities. A CAD evaluation platform is urgently needed to aid threshold selection attuned to specific contexts.

### Contributors

### Data sharing

The anonymised datasets used in this study can be made available upon reasonable request to the corresponding author. Chest x-ray images will not be provided as these are withheld by the corresponding author's organisation to reserve their use for product evaluations.

**This online publication has been corrected. The corrected version first appeared at thelancet.com/digital-health on August 21, 2024**

## Declaration of interests

CMD declares research grants from US National Institutes of Health, German Ministry of Education and Research, German Alliance for Global Health Research, United States Agency for International Development, FIND, German Center for Infection Research, and WHO. CMD also declares that she serves as an academic editor for *PLoS Medicine* and on the WHO Technical Advisory Group on tuberculosis diagnostics. All other authors declare no competing interests.
