# Correction to *Lancet Digit Health* 2024; 6: e605–13

**DOI:** 10.1016/S2589-7500(24)00176-6

**Published:** 2024-08-21

*Qin ZZ, Van der Walt M, Moyo S, et al. Computer-aided detection of tuberculosis from chest radiographs in a tuberculosis prevalence survey in South Africa: external validation and modelled impacts of commercially available artificial intelligence software.* Lancet Digit Health *2024; **6:** e605–13*—In figure 2B of this Article, the x-axis scale should have read “1·0”. This correction has been made as of Aug 21, 2024.

