# Supplementary material for: Computer-aided detection of tuberculosis from chest radiographs in a tuberculosis prevalence survey in South Africa: external validation and modelled impacts of commercially available artificial intelligence software
Source: Lancet Digit Health. 2024 Jul 19;6(9):e605–13. doi: 10.1016/S2589-7500(24)00118-3 (PMC11339183; doi:10.1016/S2589-7500(24)00118-3)
Supplement: Supplementary appendix [file mmc1.pdf]

THE LANCET
Digital Health

Supplementary appendix

This appendix formed part of the original submission and has been peer reviewed. We post it as supplied by the authors.

Supplement to: Qin ZZ, Van der Walt M, Moyo S, et al. Computer-aided detection of tuberculosis from chest radiographs in a tuberculosis prevalence survey in South Africa: external validation and modelled impacts of commercially available artificial intelligence software. *Lancet Digit Health* 2024; published online July 19. https://doi.org/10.1016/S2589-7500(24)00118-3.

ANNEXES

Contents

ANNEXES 1

Annex 1: Modeled population calculations 2

Annex 2: South African prevalence survey collection methods 2

Annex 3. Multivariable analysis 3

Annex 4: CAD products, software version and companies included in this analysis. 12

Annex 5: Histograms of CAD score for each product by bacteriologically confirmed status and history of TB 13

Annex 6. The Areas Under the Curve (AUC) of the Receiver Operated Curves (ROC) of 12 commercially available artificial intelligence products compared to the composite, culture-only and Xpert -only reference standards. 14

Annex 7: P-values of differences between CAD products. Products with significant difference (<0.0042) are highlighted in green. 15

Annex 8: Threshold Score Selection 16

Annex 9. Dynamics between sensitivity, abnormality score, abnormal CXR rate, confirmation test saving and positive rate. 19

Annex 10. The AUCs and the p-value of different subgroups 20

Annex 11: Sensitivity and Specificity for each CAD in each subgroup at a threshold of 0.5 25

Annex 12: Different CAD software specificity and threshold at pre-defined sensitivity levels and disaggregated by age groups, HIV status and prior TB history. 35

Annex 13: Sensitivity and specificity of each CAD across the entire threshold abnormality score range in age (young age [15<35 years], middle age [35<55 years], and old age [55+ years]), HIV, and prior TB history subgroups. 38

Annex 1: Modeled population calculations

Pop_{size} denotes the size of the hypothetical population, $XR_{Abnormal}$ denotes the proportion of people whose chest radiographs are deemed abnormal by the AI software (above the certain threshold score). $Xpert_{Saved}$ denotes the proportion of Xpert saved. PPV denotes positive predictive value, NPV denotes negative predictive value. P_H denotes the prevalence of the hypothetical population. We used the following formula to calculate the parameters at each threshold from 0 to 1 by an increment of 0.01 each time.

$$PPV = \frac{sensitivity \times P_H}{(sensitivity \times P_H) + (1 - specificity)(1 - P_H)}$$

$$NPV = \frac{specificity \times (1 - P_H)}{(specificity \times (1 - P_H)) + (1 - sensitivity)P_H}$$

CAD_{Pos_TB} is computed as in the case-control sample, the number of individuals whose AI score is above a certain threshold score, and the true outcome based on the reference standard is TB divided by the number of individuals whose true outcome is based on the reference standard is TB.

CAD_{Pos_normal} is computed as in the case-control sample, the number of individuals whose AI score is above a certain threshold score, and the true outcome based the reference standard is NOT TB divided by the number of individuals whose true outcome based the reference standard is NOT TB

$$XR_{Abnormal} = \frac{Pop_{size} \times P_H \times CAD_{Pos\_TB} + (1 - Pop_{size}) \times P_H \times CAD_{Pos\_normal}}{Pop_{size}}$$

$$Xpert_{Saved} = 1 - XR_{Abnormal}$$

Annex 2: South African prevalence survey collection methods

In the South African prevalence survey, socio-demographic characteristics, other TB-related data, medical conditions, smoking status, and self-reported HIV status were also collected. HIV testing by dried blood spots was also offered to those who screened positive on symptoms or CXR. The final HIV status was based on the dried blood spot result where available, and on self-reported status for those who declined testing. HIV status was categorized as missing if status was not self-reported and testing was declined.

Annex 3. Multivariable analysis

Methods

Multivariable analysis was conducted to investigate the impact of demographic and clinical variables in improving the prediction of each of the 12 CAD software. For each CAD, a multivariable logistic regression model was defined containing variables representing CAD score, age, sex, symptoms, smoking, HIV, TB history, and region. After testing variable associations using Pearson Product-Moment correlation an additional term to incorporate a sex-smoking interaction was included. Adjusted odds ratios (adjusted ORs) and p-values were then calculated for all model variables.

Multivariable model AUC was calculated for all CAD using 5-fold cross validation due to the limited sample size. In addition, as in prior analyses, a threshold score was then chosen for each model to ensure performance at 90% sensitivity and 70% specificity in turn and the sensitivity, specificity, confirmation tests saved, and yield was calculated for the hypothetical 9,000-population to compare each multivariable model to using CAD alone as a triage test.

To test how the performance of multivariable models varies with differing combinations of clinical and demographic variables, we stratified the population by their age group, HIV status, TB history and smoking status, and calculated the model AUC, sensitivity, and specificity (at the 90% threshold) in each stratum of the population. This was done for the CAD with the highest- and lowest-performing multivariable model.

Results

Variable analysis

For all multivariable models, CAD score had the greatest influence on prediction with adjusted OR’s markedly higher than any other variable and all with $p<0.0005$ (Table 3A). However, the adjusted OR varied widely across CAD from 3.89 for RADIFY- also, the CAD with the lowest AUC- to 4631 for Xvision. Despite lower adjusted ORs, age and TB history also had a significant impact on predictions for all CAD except Lunit and TiSepX TB (for the former) and CAD4TB and Genki (for the latter). Variables that were occasionally significant in models included: HIV status ($p<0.005$ for ChestEye and InferRead; $p<0.05$ for Lunit and Xvision), symptom status ($p<0.005$ for RADIFY; $p<0.005$ for XrayAME), and region ($p<0.05$ for Lunit).

Table 3A. Multivariable model summary for each CAD product

**Model Variable - Adjusted OR**	**CAD4TB**	**ChestEye**	**Genki**	**InferRead**	**JF CXR-2**	**Lunit INSIGHT CXR**	**Nexus**	**qXR**	**RADIFY**	**TiSepX-TB**	**XrayAME**	**Xvision**
Intercept	-2.22 **	-2.26 **	-2.17 **	-3.97 ***	-2.22 **	-3.00 ***	-4.03***	-2.51**	-0.266	-2.27**	-1.62*	-3.22***
CAD score	**86.9*** **	**1844*** **	**120*** **	**732*** **	**88.4*** **	**161*** **	**734*** **	**134*** **	**3.89*** **	**173*** **	**39.8*** **	**4631*** **
Age	**0.986***	**0.979** **	**0.983***	**0.980** **	**0.972*** **	0.994	**0.974** **	**0.976** **	**0.978*** **	0.997	**0.977*** **	**0.982***
Sex	1.5	1.56	1.58	1.84	1.33	1.23	1.43	1.27	0.866	0.665	2	1.98
Symptomatic	0.788	0.935	0.904	0.976	1.26	0.989	1.17	1.23	**0.538*** **	0.683	**0.627** **	0.867
Smoking	0.765	0.611	0.872	1.02	0.647	0.376	0.595	0.597	0.785	0.474	0.774	0.803
HIV	1.46	**2.15** **	1.53	**2.08** **	1.44	**1.83***	1.22	**1.80***	1.53	1.66	1.67	**1.94***
TB history	0.685	**0.484***	0.671	**0.566***	**0.542***	**0.505***	**0.589***	**0.461** **	**1.75** **	**2.06** **	**0.861** **	**0.538***
Region=Rural	1.49	1.33	1.23	1.38	1.29	**1.57***	1.37	1.42	1.28	1.22	0.935	1.33
Sex・Smoking	1.29	1.2	1.12	0.929	1.33	1.83	1.23	1.33	1.51	1.98	1.24	1.01

Significant values are in **bold,** where * = p<0.05; ** = p<0.005; *** = p<0.0005

Multivariable model performance

For most CAD, multivariable models improved the AUC compared to using CAD alone – this was true for ChestEye, InferRead, JF CXR-2, RADIFY, TiSepX-TB, XrayAME, and Xvision. The highest gain was observed for RADIFY from 0.629 (95% CI: 0.588-0.670) to 0.704 (95% CI: 0.623-0.785). In contrast, no improvement in AUC was observed for CAD4TB, Genki, Lunit, Nexus, and qXR. For CAD4TB and Lunit, AUC notably decreased from 0.850 (95% CI: 0.821-0.879) to 0.836 (95% CI: 0.768-0.903) and from 0.902 (95% CI:0.879-0.926) to 0.892 (95% CI: 0.839-0.945), respectively. However, confidence intervals overlapped for all.

At 90% sensitivity, specificity improved when utilizing the multivariable approach for most CAD: ChestEye, Genki, InferRead, JF CXR-2, Lunit, Nexus, RADIFY, XrayAME, Xvision (Tables 3B). The estimated increase in specificity was greatest for RADIFY (+16.2%) and lowest for Nexus (+1.8%). However, only the multivariable models of Lunit and ChestEye had specificity point estimates surpassing the WHO target value of 70%. For CAD4TB, qXR, and TiSepX-TB, specificity was reduced when using the multivariable model, although this was only notable for CAD4TB where specificity declined from 55.7% to 43.9%, saving fewer follow-on diagnostic tests.

At 70% specificity, the multivariable model improved sensitivity for all CAD, with the greatest difference seen for RADIFY (+22.2%) and the lowest for Nexus (+1%), increasing yield while utilizing a similar number of diagnostic tests. However, model sensitivity only surpassed the WHO target value of 90% for ChestEye and Lunit.

Tables 3B (1-12). Multivariable model AUC, sensitivity, specificity, tests saved, and yield at TPP values for each CAD product.

1. CAD4TB

- CAD4TB only: AUC = 0.850 (95% CI: 0.821-0.879)
- Multivariable model: AUC = 0.836 (95% CI: 0.768-0.903)

TPP Target	Model	Threshold score	Sensitivity	Specificity	Tests saved (%)	TB yield (%)
**No triage test**						180
**90% Sensitivity**	CAD4TB only	3	89.9%	55.7%	4676 (52%)	162 (90%)
	Multivariable	12	90.2%	43.9%	3890 (43%)	162 (90%)
**70% Specificity**	CAD4TB only	12	81.0%	70.6%	6209 (69%)	147 (82%)
	Multivariable	18	83.7%	69.9%	6190 (69%)	151 (84%)

2. ChestEye

- ChestEye only: AUC = 0.855 (95% CI: 0.827-0.883)
- Multivariable model: AUC= 0.866 (95% CI:0.793-0.939)

TPP Target	Model	Threshold score	Sensitivity	Specificity	Tests saved (%)	TB yield (%)
**No triage test**						180
**90% Sensitivity**	ChestEye only	0.08	89.1%	61.3%	5307 (59%)	162 (90%)
	Multivariable	0.17	90.2%	70.8%	6270 (70%)	162 (90%)
**70% Specificity**	ChestEye only	0.11	86.0%	69.6%	6201 (69%)	155 (86%)
	Multivariable	0.16	91.2%	69.9%	6180 (69%)	164 (91%)

3. Genki

- Genki only: AUC = 0.847 (95% CI: 0.817-0.876)
- Multivariable model: AUC= 0.846 (95% CI: 0.773-0.919)

TPP Target	Model	Threshold score	Sensitivity	Specificity	Tests saved (%)	TB yield (%)
No triage test						180
**90% Sensitivity**	Genki only	0.02	89.9%	54.5%	4641 (52%)	162 (90%)

	Multivariable	0.14	90.2%	57.8%	5120 (57%)	162 (90%)
**70% Specificity**	Genki only	0.09	84.5%	70.6%	6204 (69%)	156 (87%)
	Multivariable	0.17	87.0%	69.9%	6190 (69%)	157 (87%)

4. InferRead DR Chest

- InferRead only: AUC = 0.854 (95% CI: 0.824- 0.884)
- Multivariable model: AUC= 0.872 (95% CI: 0.812-0.933)

TPP Target	Model	Threshold score	Sensitivity	Specificity	Tests saved (%)	TB yield (%)
**No triage test**						180
**90% Sensitivity**	InferRead only	0.26	90.3%	54.9%	4880 (54%)	162 (90%)
	Multivariable	0.14	90.2%	64.1%	5670 (63%)	162 (90%)
**70% Specificity**	InferRead only	0.37	85.7%	70.0%	6202 (69%)	154 (86%)
	Multivariable	0.18	89.8%	69.9%	6180 (69%)	162 (90%)

5. JF CXR

- JF CXR only: AUC = 0.865 (95% CI: 0.839-0.892)
- Multivariable model: AUC= 0.867 (95% CI: 0.802-0.931)

TPP Target	Model	Threshold score	Sensitivity	Specificity	Tests saved (%)	TB yield (%)
**No triage test**						180
**90% Sensitivity**	JF CXR only	0.23	89.5%	62.7%	5259 (58%)	162 (90%)
	Multivariable	0.14	90.2%	68.2%	6030 (67%)	162 (90%)
**70% Specificity**	JF CXR only	0.40	86.4%	70.0%	6199 (69%)	156 (87%)
	Multivariable	0.17	88.8%	69.9%	6180 (69%)	160 (89%)

6. Lunit INSIGHT CXR

- Lunit INSIGHT CXR only: AUC = 0.902 (95% CI: 0.879-0.926)
- Multivariable model: AUC= 0.892 (95% CI: 0.839-0.945)

TPP Target	Model	Threshold score	Sensitivity	Specificity	Tests saved (%)	TB yield (%)
**No triage test**						180
**90% Sensitivity**	Lunit INSIGHT CXR only	0.07	89.9%	67.7%	5989 (67%)	162 (90%)
	Multivariable	0.12	90.2%	76.1%	6730 (75%)	162 (90%)
**70% Specificity**	Lunit INSIGHT CXR only	0.09	89.5%	70.2%	6195 (69%)	161 (89%)
	Multivariable	0.10	91.2%	69.9%	6180 (69%)	164 (91%)

7. Nexus

- Nexus only: AUC = 0.897 (95% CI: 0.872-0.922)
- Multivariable model: AUC= 0.896 (95% CI: 0.827-0.964)

TPP Target	Model	Threshold score	Sensitivity	Specificity	Tests saved (%)	TB yield (%)
**No triage test**		-	-	-	-	180
**90% Sensitivity**	Nexus only	0.48	89.9%	67.1%	5938 (66%)	162 (90%)
	Multivariable	0.20	90.2%	68.9%	6100 (68%)	162 (90%)
**70% Specificity**	Nexus only	0.54	88.8%	69.8%	6179 (69%)	160 (89%)
	Multivariable	0.21	89.8%	69.9%	6180 (69%)	162 (90%)

8. qXR

- qXR only: AUC = 0.878 (95% CI: 0.853-0.904)
- qXR multivariable : AUC = 0.876 (95% CI: 0.823-0.930)

TPP Target	Model	Threshold score	Sensitivity	Specificity	Tests saved (%)	TB yield (%)
**No triage test**		-	-	-	-	180 (100%)
**90% Sensitivity**	qXR only	0.18	90.3%	62.3%	5511 (61%)	162 (90%)
	Multivariable	0.13	90.2%	60.2%	5330 (59%)	162 (90%)
**70% Specificity**	qXR only	0.32	86.8%	70.2%	6216 (69%)	157 (87%)
	Multivariable	0.14	89.8%	69.9%	6180 (69%)	162 (90%)

9. RADIFY

- RADIFY only: AUC = 0.629 (95% CI: 0.588- 0.670)
- RADIFY multivariable: AUC= 0.704 (95% CI: 0.623-0.785)

TPP Target	Model	Threshold score	Sensitivity	Specificity	Tests saved (%)	TB yield (%)
**No triage test**		-	-	-	-	180
**90% Sensitivity**	RADIFY only	0.02	82.6%$^{+}$	32.5%	2897 (32%)	149 (83%)
	Multivariable*	0.26	82.8%*	48.7%	4320 (48%)	149 (83%)
**70% Specificity**	RADIFY only	0.57	45.7%	69.4%	6222 (69%)	82 (46%)
	Multivariable	0.34	67.9%	69.9%	6220 (69%)	122 (68%)

$^{+}$ closest to 90% sensitivity;
*****set to match the sensitivity achieved by the product alone in the initial TPP analysis.

10. TiSepX- TB

- TiSepX TB only: AUC = 0.821 (95% CI: 0.787-0.854)
- TiSepX TB multivariable model: AUC= 0.838 (95% CI: 0.725-0.951)

TPP Target	Model	Threshold score	Sensitivity	Specificity	Tests saved (%)	TB yield (%)
**No triage test**	-	-	-	-		180
**90% Sensitivity**	TiSepX TB only	0.18	89.9%	48.0%	4198 (47%)	162 (90%)
	Multivariable	0.14	90.2%	47.0%	4160 (46%)	162 (90%)
**70% Specificity**	TiSepX TB only	0.29	77.5%	70.8%	6215 (69%)	140 (78%)
	Multivariable	0.21	84.7%	69.9%	6190 (69%)	152 (84%)

11. XrayAME

- XrayAME only: AUC = 0.762 (95% CI: 0.725 – 0.799)
- XrayAME Multivariable model: AUC= 0.795 (95% CI: 0.754-0.836)

TPP Target	Model	Threshold score	Sensitivity	Specificity	Tests saved (%)	TB yield (%)
**No triage test**	-	-	-	-		180
**90% Sensitivity**	XrayAME only	0.02	88.4%	36.9%	3004 (33%)	162 (90%)
	Multivariable	0.17	90.2%	45.5%	4030 (45%)	162 (90%)
**70% Specificity**	XrayAME only	0.13	68.6%	70.6%	6232 (69%)	123 (68%)
	Multivariable	0.25	78.6%	69.9%	6200 (69%)	141 (78%)

12. Xvision

- Xvision only: AUC = 0.861 (95% CI: 0.833– 0.890)
- Xvision multivariable model: AUC= 0.872 (95% CI: 0.824-0.920)

TPP Target	Model	Threshold score	Sensitivity	Specificity	Tests saved (%)	TB yield (%)
**No triage test**	-	-	-	-		180
**90% Sensitivity**	Xvision only	0.11	89.9%	58.6%	5138 (57%)	162 (90%)
	Multivariable	0.15	90.2%	68.2%	6030 (67%)	162 (90%)
**70% Specificity**	Xvision only	0.14	85.7%	69.6%	6202 (69%)	154 (86%)
	Multivariable	0.16	88.8%	69.9%	6180 (69%)	160 (89%)

Population strata analysis

Strata analysis results are presented for RADIFY and Nexus as our study sample size is limited and under powered. RADIFY's multivariable model exhibited a wide range of AUCs across strata, from 0.452 (95% CI: 0.084-0.452) in middle-aged non-smokers without HIV and with prior TB, to 0.900 (95% CI: 0.623-0.900) in young non-smokers with HIV and prior TB. The AUC in RADIFY's top-performing stratum outperformed other demographics, including the lowest-performing stratum, the older-aged group with the same characteristics, young smokers with the same clinical indications, middle- and older-aged non-smokers with no clinical conditions, older non-smokers with HIV but no prior TB, as well as middle-aged smokers with HIV and no prior TB. For the lowest-performing stratum, AUC was lower than that of the older population with the same characteristics and young- and middle-aged smokers with the same clinical indications, as well as some other demographics (Table 3C-A).

Sensitivity ranged from 26.3% (95% CI: 6.50 – 46.1%) in older non-smokers without HIV nor prior TB, to 100% (95% CI: 100 – 100%) in multiple strata (Table 3C-A). Compared to the overall sensitivity of 82.8%, performance was significantly worse in middle- and old-aged non-smokers with no clinical indications. Furthermore, holding all other variables the same, sensitivity declined between young and old age groups across strata, but this was only significant in HIV-negative, non-smokers without prior TB.

Specificity varied from 0.00% (95% CI: 0.00 – 0.00%) in multiple strata (Table 3C-A), to 87.0% (95% CI: 80.1-93.8%) in older non-smokers without HIV nor prior TB. Compared to the overall specificity of 48.7%, performance was lower in smokers with prior TB and without HIV regardless of age, in young HIV-negative non-smokers with prior TB, in young and middle-aged individuals with HIV and prior TB regardless of smoking, and in young smokers without other clinical indications. Many strata with prior TB had 0% specificity and, in general, prior TB was associated with reduced performance. This was significant for young HIV-negative individuals regardless of smoking status, older HIV-negative smokers, and middle-aged HIV-positive non-smokers. In contrast, in two strata specificity improved upon the overall estimate. These were middle- and old-aged non-smokers without clinical indications, with specificities of 65.9% (95% CI: 51.3 – 80.4%) and 87.0% (95% CI: 80.1 – 93.8%), respectively.

Meanwhile, Nexus' multivariable model demonstrated AUCs ranging from 0.548 (95% CI: 0.189 - 0.548) in middle aged smokers with HIV and prior TB, to 1.00 (95% CI: 1.00-1.00) in numerous strata (Table 3C-B). The former was significantly lower than all others, except young smokers without prior TB and with HIV as well as old non-smokers without HIV and with prior TB, where AUC was 0.829 (95% CI: 0.547 - 0.829) and 0.726 (95% CI: 0.386 - 0.726), respectively. The lowest AUC for Nexus was significantly better than that for RADIFY, which performed worse than random chance.

For Nexus, sensitivity was lowest in middle-aged smokers with HIV and prior TB (57.1% [95% CI: 20.5-93.8%]), and highest in several strata that achieved 100% (Table 3C-B). Only one stratum had a confidence interval that did not cross the 90% sensitivity value: old, HIV-negative, non-smokers without prior TB, where sensitivity was 68.4% (95% CI: 47.5 - 89.3%), although point estimates were <90% in several others (Table 3-B).

Specificity was lowest in young, non-smokers, without HIV but with prior TB (0.00% [95% CI: 0.00 – 0.00%]) and highest in young, non-smokers, without prior TB who were HIV-positive (100% [95% CI: 100 – 100%]), where a

perfect classification was achieved. Specificities lower than the overall estimate of 68.9% were entirely concentrated in those with prior TB, including in middle-aged HIV-positive smokers, in middle- and older- aged HIV-positive non-smokers, in HIV-negative smokers regardless of age, and in young non-smokers without HIV. In contrast, several strata had specificity > 68.9%, none of which had TB history. These included young and middle-aged HIV-positive non-smokers, young HIV negative smokers and non-smokers, and old non-smokers who are HIV negative.

Table 3C. Performance of the multivariable model in different population strata for A) RADIFY and B) Nexus.

A) RADIFY

Age Group	Smoking	HIV Status	TB History	AUC (95% CI)	Sensitivity (95% CI)	Specificity (95% CI)
Young	Smoker	Positive	None	0.657 (95% CI: 0.295 - 0.657)	**100% (95% CI: 100 – 100%)**	**0.00% (95% CI: 0.00 – 0.00%)**
Middle	Smoker	Positive	None	0.580 (95% CI: 0.295 - 0.580)	**100% (95% CI: 100 – 100%)**	22.2% (95% CI: 0.00 – 49.4%)
Old	Smoker	Positive	None	NA*	NA*	NA*
Young	Non-smoker	Positive	None	0.857 (95% CI: 0.611 - 0.857)	**100% (95% CI: 100 – 100%)**	**0.00% (95% CI: 0.00 – 0.00%)**
Middle	Non-smoker	Positive	None	0.922 (95% CI: 0.801 - 0.922)	88.9% (95% CI: 68.4% - 100%)	60.0% (95% CI: 29.6 – 90.4%)
Old	Non-smoker	Positive	None	NA*	NA*	NA*
Young	Smoker	Negative	None	0.771 (95% CI: 0.652 - 0.771)	95.5% (95% CI: 86.8 – 100%)	26.5% (95% CI: 14.2 – 38.9%)
Middle	Smoker	Negative	None	0.703 (95% CI: 0.558 - 0.703)	80.0% (95% CI: 64.3 – 95.7%)	48.3% (95% CI: 30.1 – 66.5%)
Old	Smoker	Negative	None	0.705 (95% CI: 0.482 - 0.705)	75.0% (95% CI: 50.5 – 99.5%)	61.8% (95% CI: 45.4 – 78.1%)
Young	Non-smoker	Negative	None	0.705 (95% CI: 0.563 - 0.705)	72.2% (95% CI: 51.5 – 92.9%)	51.2% (95% CI: 35.9 – 66.5%)
Middle	Non-smoker	Negative	None	0.603 (95% CI: 0.344 - 0.603)	42.9% (95% CI: 6.20 – 79.5%)	65.9% (95% CI: 51.3 – 80.4%)
Old	Non-smoker	Negative	None	0.573 (95% CI: 0.415 - 0.573)	**26.3% (95% CI: 6.50 – 46.1%)**	**87.0% (95% CI: 80.1 – 93.8%)**
Young	Smoker	Positive	Prior TB	0.600 (95% CI: 0.00 - 0.600)	**100% (95% CI: 100 – 100%)**	**0.00% (95% CI: 0.00 – 0.00%)**
Middle	Smoker	Positive	Prior TB	0.440 (95% CI: 0.127 - 0.440)	**100% (95% CI: 100 – 100%)**	**0.00% (95% CI: 0.00 – 0.00%)**
Old	Smoker	Positive	Prior TB	NA*	NA*	NA*
Young	Non-smoker	Positive	Prior TB	**0.900 (95% CI: 0.623 - 0.900)**	**100% (95% CI: 100 – 100%)**	**0.00% (95% CI: 0.00 – 0.00%))**
Middle	Non-smoker	Positive	Prior TB	0.604 (95% CI: 0.379 - 0.604)	**100% (95% CI: 100 – 100%)**	**0.00% (95% CI: 0.00 – 0.00%)**
Old	Non-smoker	Positive	Prior TB	0.607 (95% CI: 0.153 - 0.607)	50.0% (95% CI: 1.00% - 99.0%)	28.6% (95% CI: 0.00 – 62.0%)
Young	Smoker	Negative	Prior TB	0.758 (95% CI: 0.518 - 0.758)	**100% (95% CI: 100 – 100%)**	**0.00% (95% CI: 0.00 – 0.00%)**
Middle	Smoker	Negative	Prior TB	0.836 (95% CI: 0.670 - 0.836)	**100% (95% CI: 100 – 100%)**	20.0% (95% CI: 0.00 – 44.8%)
Old	Smoker	Negative	Prior TB	0.714 (95% CI: 0.381 - 0.714)	75.0% (95% CI: 32.6% - 100%)	14.3% (95% CI: 0.00 – 40.2%)
Young	Non-smoker	Negative	Prior TB	0.750 (95% CI: 0.234 - 0.750)	**100% (95% CI: 100 – 100%)**	**0.00% (95% CI: 0.00 – 0.00%)**
Middle	Non-smoker	Negative	Prior TB	**0.452 (95% CI: 0.084 - 0.452)**	85.7% (95% CI: 59.8 – 100%)	33.3% (95% CI: 0.00 – 71.1%)
Old	Non-smoker	Negative	Prior TB	0.705 (95% CI: 0.478 - 0.705)	80.0% (95% CI: 44.9 – 100%)	57.9% (95% CI: 35.7 – 80.1%)

*NA indicates there was not enough data in this stratum to complete the analysis
For each analysis, lowest and highest values are **bold.** For sensitivity and specificity analyses, cells in **red** indicate lower performance than the value achieved by the multivariable overall, cells in **green** indicate a higher performance.

B) Nexus

Age Group	Smoking	HIV Status	TB History	AUC (95% CI)	Sensitivity (95% CI)	Specificity (95% CI)
Young	Smoker	Positive	None	0.829 (95% CI: 0.547 - 0.829)	85.7% (95% CI: 59.792 – 100%)	80.0% (95% CI: 44.9 – 100%)
Middle	Smoker	Positive	None	**1.00 (95% CI: 1.00 – 1.00)**	**100% (95% CI: 100 – 100%)**	55.6% (95% CI: 23.1 - 88.0%)
Old	Smoker	Positive	None	NA*	NA*	NA*
Young	Non-smoker	Positive	None	**1.00 (95% CI: 1.00 – 1.00)**	**100% (95% CI: 100 – 100%)**	**100% (95% CI: 100 – 100%)**
Middle	Non-smoker	Positive	None	0.922 (95% CI: 0.797 - 0.922)	88.9% (95% CI: 68.4 – 100%)	90.0% (95% CI: 71.4 – 100%)
Old	Non-smoker	Positive	None	NA*	NA*	NA*
Young	Smoker	Negative	None	0.965 (95% CI: 0.906 - 0.965)	95.5% (95% CI: 86.8 – 100%)	81.6% (95% CI: 70.8 - 92.5%)
Middle	Smoker	Negative	None	0.948 (95% CI: 0.894 - 0.948)	**100% (95% CI: 100 – 100%)**	72.4% (95% CI: 56.2 - 88.7%)
Old	Smoker	Negative	None	0.887 (95% CI: 0.735 - 0.887)	91.7% (95% CI: 76.0 – 100%)	70.6% (95% CI: 55.3 - 85.9%)
Young	Non-smoker	Negative	None	0.867 (95% CI: 0.741 - 0.867)	83.3% (95% CI: 66.1 – 100%)	82.9% (95% CI: 71.4 - 94.4%)
Middle	Non-smoker	Negative	None	0.916 (95% CI: 0.823 - 0.916)	85.7% (95% CI: 59.8 – 100%)	80.5% (95% CI: 68.4 - 92.6%)
Old	Non-smoker	Negative	None	0.847 (95% CI: 0.74 - 0.847)	68.4% (95% CI: 47.5 - 89.3%)	82.6% (95% CI: 74.9 - 90.4%)
Young	Smoker	Positive	Prior TB	**1.00 (95% CI: 1.00 – 1.00)**	**100% (95% CI: 100 – 100%)**	50.0% (95% CI: 0.00 – 100%)
Middle	Smoker	Positive	Prior TB	**0.548 (95% CI: 0.189 - 0.548)**	**57.1% (95% CI: 20.5 - 93.8%)**	16.7% (95% CI: 0.00 - 37.8%)
Old	Smoker	Positive	Prior TB	NA*	NA*	NA*
Young	Non-smoker	Positive	Prior TB	**1.00 (95% CI: 1.00 – 1.00)**	**100% (95% CI: 100 – 100%)**	50.0% (95% CI: 0.00 – 100%)
Middle	Non-smoker	Positive	Prior TB	0.802 (95% CI: 0.630 - 0.802)	92.9% (95% CI: 79.4 – 100%)	23.1% (95% CI: 0.174 – 46.0%)
Old	Non-smoker	Positive	Prior TB	0.821 (95% CI: 0.46 - 0.821)	75.0% (95% CI: 32.6 – 100%)	28.6% (95% CI: 0.00 - 62.0%)
Young	Smoker	Negative	Prior TB	0.912 (95% CI: 0.77 - 0.912)	**100% (95% CI: 100 – 100%)**	14.3% (95% CI: 0.00 - 40.2%)
Middle	Smoker	Negative	Prior TB	0.793 (95% CI: 0.595 - 0.793)	**100% (95% CI: 100 – 100%)**	20.0% (95% CI: 0.00% - 44.8%)
Old	Smoker	Negative	Prior TB	0.857 (95% CI: 0.56 - 0.857)	**100% (95% CI: 100 – 100%)**	28.6% (95% CI: 0.00 - 62.0%)
Young	Non-smoker	Negative	Prior TB	**1.00 (95% CI: 1.00 – 1.00)**	**100% (95% CI: 100 – 100%)**	**0.00% (95% CI: 0.00 – 0.00%)**
Middle	Non-smoker	Negative	Prior TB	0.976 (95% CI: 0.91 - 0.976)	**100% (95% CI: 100 – 100%)**	33.3% (95% CI: 0.00 - 71.1%)
Old	Non-smoker	Negative	Prior TB	0.726 (95% CI: 0.386 - 0.726)	60.0% (95% CI: 17.1 – 100%)	63.2% (95% CI: 41.5 - 84.9%)

*NA indicates there was not enough data in this stratum to complete the analysis
For each analysis, lowest and highest values are **bold.** For sensitivity and specificity analyses, cells in **red** indicate lower performance than the value achieved by the multivariable overall, cells in **green** indicate a higher performance.

Discussion

In conclusion, the findings suggest that incorporating multiple demographic and clinical features into CAD models, alongside the CAD software, has the potential to enhance the accuracy of CAD systems and contribute to more effective medical decision-making. This is particularly beneficial for CAD products with lower overall performance.

Similar to the sub-group analysis, the multivariable regression analysis revealed that age and prior TB history consistently emerged as important variables influencing predictions in most multivariable models, in addition to

the CAD score. However, it is important to acknowledge that the impact of these variables may not be universal across all CAD systems.

Furthermore, the strata analysis identified how, even at a threshold chosen to elicit performance at a specific level, actual accuracy can vary. This intersectional approach highlighted how the complex interactions of individual-level demographic and clinical factors influence performance, echoing the results of earlier analyses and literature suggesting the influence of age and TB history on CAD performance. Despite this, higher-performing CAD are suggested to be capable of retaining a good standard regardless of intersecting individual-level factors, compared to lower-performing products.

It is worth noting that the sample size in this study was relatively small, which may limit the power and generalizability of the findings. This prevented the full investigation of all population strata due to a lack of data for some variable combinations. Further research with larger sample sizes is needed to validate and extend these results. Additionally, future studies should explore more advanced predictive models that incorporate a wider range of variables to capture more nuanced patterns and further improve the predictive accuracy of CAD systems.

Overall, the findings highlight the potential benefits of incorporating demographic and clinical features into CAD models, but further research is necessary to fully explore and optimize the use of these variables in CAD systems.

Annex 4: CAD products, software version and companies included in this analysis.

CAD Product Name	Software Version	CAD Company Name	CAD Company Headquarter	Abnormality score range
**CAD4TB**	version7	Delft Imaging Systems	Netherlands	0-100
**Chest Eye**	version2.4	Oxipit.ai	Lithuania	0-1
**Genki**	version 20.12	DeepTek	India	0-1
**InferRead DR Chest**	version1	Infervision	China	0-1
**JF CXR-2**	version2	JF Healthcare	China	0-1
**Lunit INSIGHT CXR**	version 4.9	Lunit	South Korea	0-1
**Nexus CXR (Google Research Model)**	version 1	Nexus	South Africa	0-1
**qXR**	version3	Qure.ai	India	0-1
**RADIFY**	version 3.5.0c	Envisionit	South Africa	0-1
**TiSepX TB**	version1.0.0.0	Medical IP	South Korea	0-1
**XrayAME**	version1	Epcon	Belgium	0-1
**XVision**	version2.2.211	Mindfully Technologies	Romania	0-1

Annex 5: Histograms of CAD score for each product by bacteriologically confirmed status and history of TB

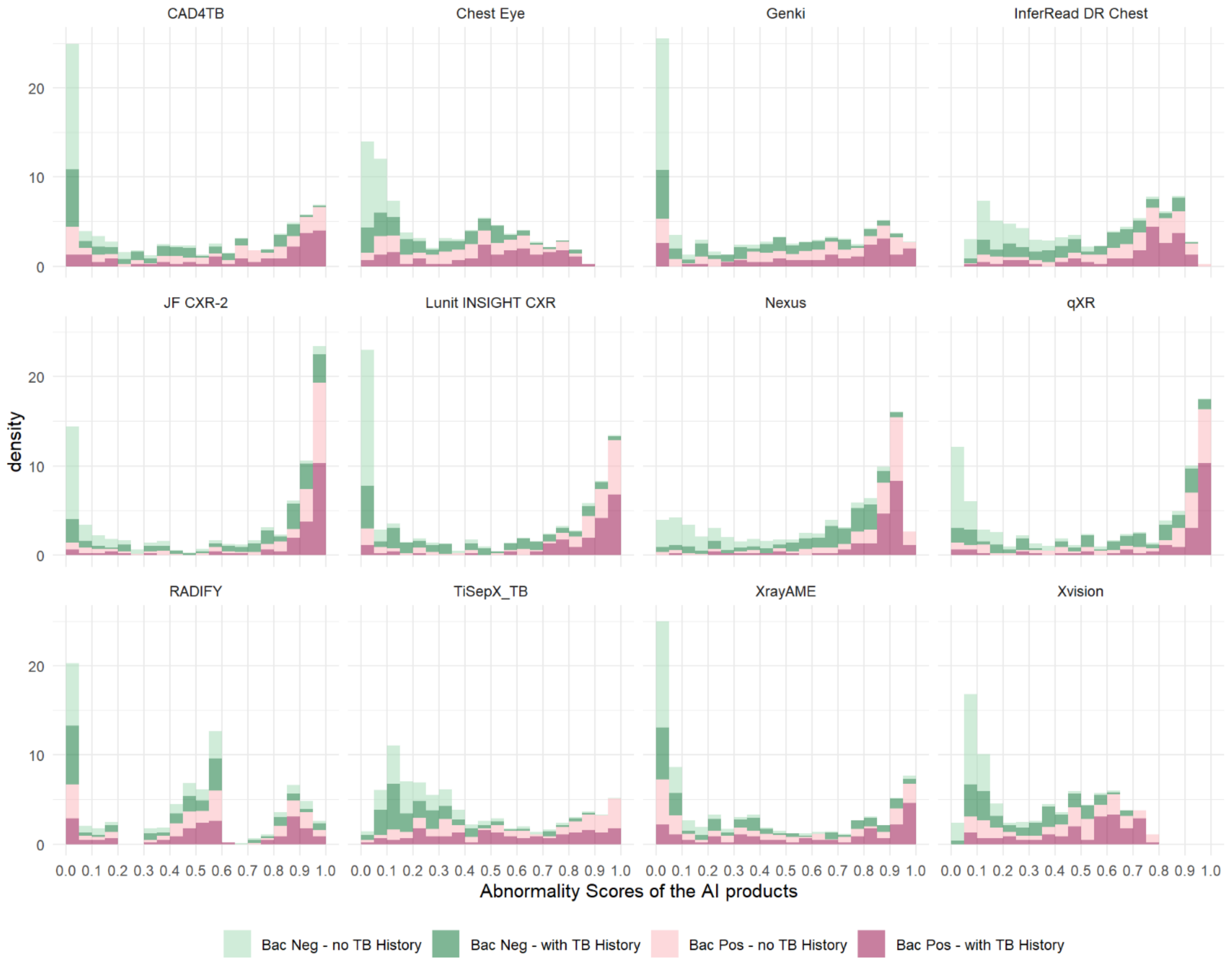

Annex 6. The Areas Under the Curve (AUC) of the Receiver Operated Curves (ROC) of 12 commercially available artificial intelligence products compared to the composite, culture-only and Xpert -only reference standards.

AUC (95% CI)	Composite MRS	Reference standard: Culture only	Reference standard: Xpert only
Lunit	0.902 (0.879-0.926)	0.857 (0.825-0.889)	0.916 (0.892-0.94)
Nexus	0.897 (0.872-0.922)	0.862 (0.828-0.896)	0.910 (0.885 - 0.935)
qXR	0.878 (0.853-0.904)	0.834 (0.801-0.867)	0.897 (0.872-0.922)
JF CXR-2	0.865 (0.839-0.892)	0.823 (0.790-0.857)	0.871 (0.844-0.899)
Xvision	0.861 (0.833-0.890)	0.824 (0.787-0.86)	0.879 (0.85-0.908)
ChestEye	0.855 (0.827-0.883)	0.813 (0.779-0.847)	0.865 (0.836-0.894)
InferRead	0.854 (0.824-0.884)	0.811 (0.773-0.848)	0.864 (0.833-0.894)
CAD4TB	0.850 (0.821-0.879)	0.807 (0.769-0.846)	0.872 (0.842-0.902)
Genki	0.847 (0.817-0.876)	0.809 (0.773-0.846)	0.869 (0.840-0.897)
TiSepX TB	0.821 (0.787-0.854)	0.801 (0.758-0.844)	0.821 (0.787-0.854)
XrayAME	0.762 (0.725-0.799)	0.739 (0.693-0.784)	0.789 (0.751-0.828)
RADIFY	0.629 (0.588-0.670)	0.593 (0.544-0.643)	0.629 (0.588-0.67)

Sensitivity analysis:

736 participants had a valid Xpert result, while 667 had a valid culture result. The AUCs of CAD products using the Xpert-only reference standard were higher than those when using the composite MRS, whereas the AUCs of all products when using the culture-only reference standard were lower. Using Xpert-only as the reference standard also resulted in a higher AUC than culture-only.

Annex 7: P-values of differences between CAD products. Products with significant difference (<0.0042) are highlighted in green.

	Lunit	Nexus	qXR	JF CXR-2	Xvision	Chest Eye	CAD4 TB	Genki	InferRead	TiSepX_TB	Xray AME
**Lunit**											
**Nexus**	0.48										
**qXR**	0.0026	0.026									
**JF CXR-2**	<0.0001	0.00063	0.14								
**Xvision**	<0.0001	<0.0001	0.031	0.60							
**ChestEye**	<0.0001	<0.0001	0.011	0.22	0.42						
**CAD4TB**	<0.0001	<0.0001	0.0036	0.17	0.22	0.63					
**Genki**	<0.0001	<0.0001	0.0011	0.096	0.14	0.43	0.71				
**InferRead**	<0.0001	<0.0001	0.0065	0.15	0.38	0.89	0.74	0.55			
**TiSepX_TB**	<0.0001	<0.0001	0.0006	0.013	0.022	0.061	0.090	0.15	0.086		
**XrayAME**	<0.0001	<0.0001	<0.0001	<0.0001	<0.0001	<0.0001	<0.0001	<0.0001	<0.0001	0.0090	
**RADIFY**	<0.0001	<0.0001	<0.0001	<0.0001	<0.0001	<0.0001	<0.0001	<0.0001	<0.0001	<0.0001	<0.0001

CONFIDENTIAL

Annex 8: Threshold Score Selection

A. Thresholds of the 12 CAD products to reach 80% sensitivity and the corresponding specificity (sorted by specificity)

Product	Threshold	Sensitivity	Specificity
Lunit	0.51	80.2% (95% CI: 74.8-84.9%)	87.8% (95% CI: 84.7-90.5%)
Nexus	0.72	79.8% (95% CI: 74.4-84.6%)	83.2% (95% CI: 79.7-86.3%)
InferRead	0.49	79.8% (95% CI: 74.4-84.6%)	81.8% (95% CI: 78.2-85.0%)
JF CXR-2	0.75	79.8% (95% CI: 74.4-84.6%)	80.3% (95% CI: 76.6-83.6%)
qXR	0.52	79.8% (95% CI: 74.4-84.6%)	77.9% (95% CI: 74.1-81.5%)
Xvision	0.18	80.6% (95% CI: 75.3-85.3%)*	77.4% (95% CI: 73.5-80.9%)
Genki	0.2	80.2% (95% CI: 74.8-84.9%)	77.4% (95% CI: 73.5 -80.9%)
ChestEye	0.14	79.8% (95% CI: 74.4-84.6%)	75.6% (95% CI: 71.7-79.3%)
CAD4TB	16	79.5% (95% CI: 74.0-84.2%)*	73.9% (95% CI: 69.9-77.6%)
TiSepX-TB	0.27	80.2% (95% CI: 74.8-84.9%)	67.9% (95% CI: 63.7-71.9%)
XrayAME	0.05	79.8% (95% CI: 74.4-84.6%)	53.2% (95% CI: 48.8-57.6%)
RADIFY	0.11	80.2% (95% CI: 74.8-84.9%)	37.9% (95% CI: 33.7-42.2%)

*The closest sensitivity to 80%

B. Thresholds of the 12 CAD products to reach 60% specificity and the corresponding sensitivity (sorted by sensitivity)

Product	Threshold	Sensitivity	Specificity
Lunit	0.04	93.0% (95% CI: 89.2-95.8%)	62.1% (95% CI: 57.8-66.3%)*

CONFI

Nexus	0.37	93.0% (95% CI: 89.2-95.8%)	60.2% (95% CI: 55.8-64.4%)
JF CXR-2	0.19	90.7% (95% CI: 86.5-93.9%)	60.5% (95% CI: 56.2-64.8%)
qXR	0.17	90.3% (95% CI: 86.0-93.6%)	60.2% (95% CI: 55.8-64.4%)
Xvision	0.11	89.9% (95% CI: 85.6-93.3%)	58.6% (95% CI: 54.2-62.9%)*
ChestEye	0.08	89.1% (95% CI: 84.7-92.7%)	61.3% (95% CI: 57.0-65.5%)
InferRead	0.29	88.4% (95% CI: 83.8-92.0%)	60.7% (95% CI: 56.4-65.0%)
CAD4TB	0.04	88.4% (95% CI: 83.8-92.0%)	58.8% (95% CI: 54.4-63.1%)*
Genki	0.03	84.4% (95% CI: 83.8-92.0%)	59.0% (95% CI: 54.6-63.3%)*
TiSepX-TB	0.23	83.3% (95% CI: 78.2-87.7%)	59.2% (95% CI: 54.8-63.5%)
XrayAME	0.07	75.2% (95% CI: 69.5-80.3%)	60.3% (95% CI: 56.0-64.6%)
RADIFY	0.50	57.8% (95% CI: 51.5-63.9%)	59.8% (95% CI: 55.4-64.0%)

*****The closest specificity to 60%

C. Thresholds to match 10% test referral rate, i.e., X-ray abnormal rate (sorted by sensitivity)

**Product**	**Threshold**	**Sensitivity**	**Specificity**
Lunit	0.69	75.2% (95% CI: 69.5-80.3%)	91.3% (95% CI: 88.5-93.6%)
Nexus	0.81	70.5% (95% CI: 64.6-76.0%)	91.1% (95% CI: 88.3-93.4%)
qXR	0.85	64.3% (95% CI: 58.2-70.2%)	91.1% (95% CI: 88.3-93.4%)
Xvision	0.43	62.8% (95% CI: 56.6-68.7%)	90.9% (95% CI: 88.1-93.2%)
InferRead	0.69	61.2% (95% CI: 55.0-67.2%)	90.9% (95% CI: 88.1-93.2%)
JF CXR-2	0.93	59.3% (95% CI: 53.0-65.4%)	91.1% (95% CI: 88.3-93.4%)

CAD4TB	0.58	59.3% (95% CI: 53.0-65.4%)	91.3% (95% CI: 88.5-93.6%)
TiSepX-TB	0.51	57.8% (95% CI: 51.5-63.9%)	90.9% (95% CI: 88.1-93.2%)
ChestEye	0.42	55.8% (95% CI: 49.5-62.0%)	90.7% (95% CI: 87.9-93.1%)
Genki	0.58	55.0% (95% CI: 48.7-61.2%)	90.7% (95% CI: 87.9-93.1%)
XrayAME	0.59	43.8% (95% CI: 37.7-50.1%)	90.7% (95% CI: 87.9-93.1%)
RADIFY	0.87	21.7% (95% CI: 16.8-27.2%)	91.1% (95% CI: 88.3-93.4%)

D. The sensitivity, specificity at threshold 0.5 (sorted by sensitivity)

**Product**	**Threshold**	**Sensitivity**	**Specificity**
Nexus	0.5	89.1% (95% CI: 84.7-92.7%)	68.3% (95% CI: 64.1-72.3%)
JF CXR-2		86.0% (95% CI: 81.2-90.0%)	71.4% (95% CI: 67.3-75.2%)
qXR		80.6% (95% CI: 75.3-85.3%)	77.2% (95% CI: 73.3-80.7%)
Lunit		80.2% (95% CI: 74.8-84.9%)	87.6% (95% CI: 84.5-90.3%)
InferRead		78.7% (95% CI: 73.2-83.5%)	82.8% (95% CI: 79.2-85.9%)
CAD4TB	50	64.0% (95% CI: 57.8-69.8%)	89.4% (95% CI: 86.4-91.9%)
Genki	0.5	61.6% (95% CI: 55.4-67.6%)	88.6% (95% CI: 85.5-91.2%)
TiSepX-TB		58.5% (95% CI: 52.3-64.6%)	90.5% (95% CI: 87.7-92.9%)
RADIFY		57.8% (95% CI: 51.5-63.9%)	59.8% (95% CI: 55.4-64.0%)
Xvision		50.4% (95% CI: 44.1-56.6%)	94.4% (95% CI: 92.0-96.2%)
XrayAME		46.9% (95% CI: 40.7-53.2%)	89.2% (95% CI: 86.2-91.7%)

CONFIDENTIAL

ChestEye		41.1% (95% CI: 35.0-47.4%)	94.2% (95% CI: 91.8-96.1%)

Annex 9. Dynamics between sensitivity, abnormality score, abnormal CXR rate, confirmation test saving and positive rate.

A) how sensitivity changes over a continuous range of thresholds, b) the abnormal CXR rate over a continuous range of thresholds, c) the trade-off between sensitivity and proportion of confirmation test saved, d) the confirmation test positivity rate over a continuous range of thresholds of the CAD product. The abnormality score of CAD4TB is converted to between 0 and 1, instead of 0 and 100.

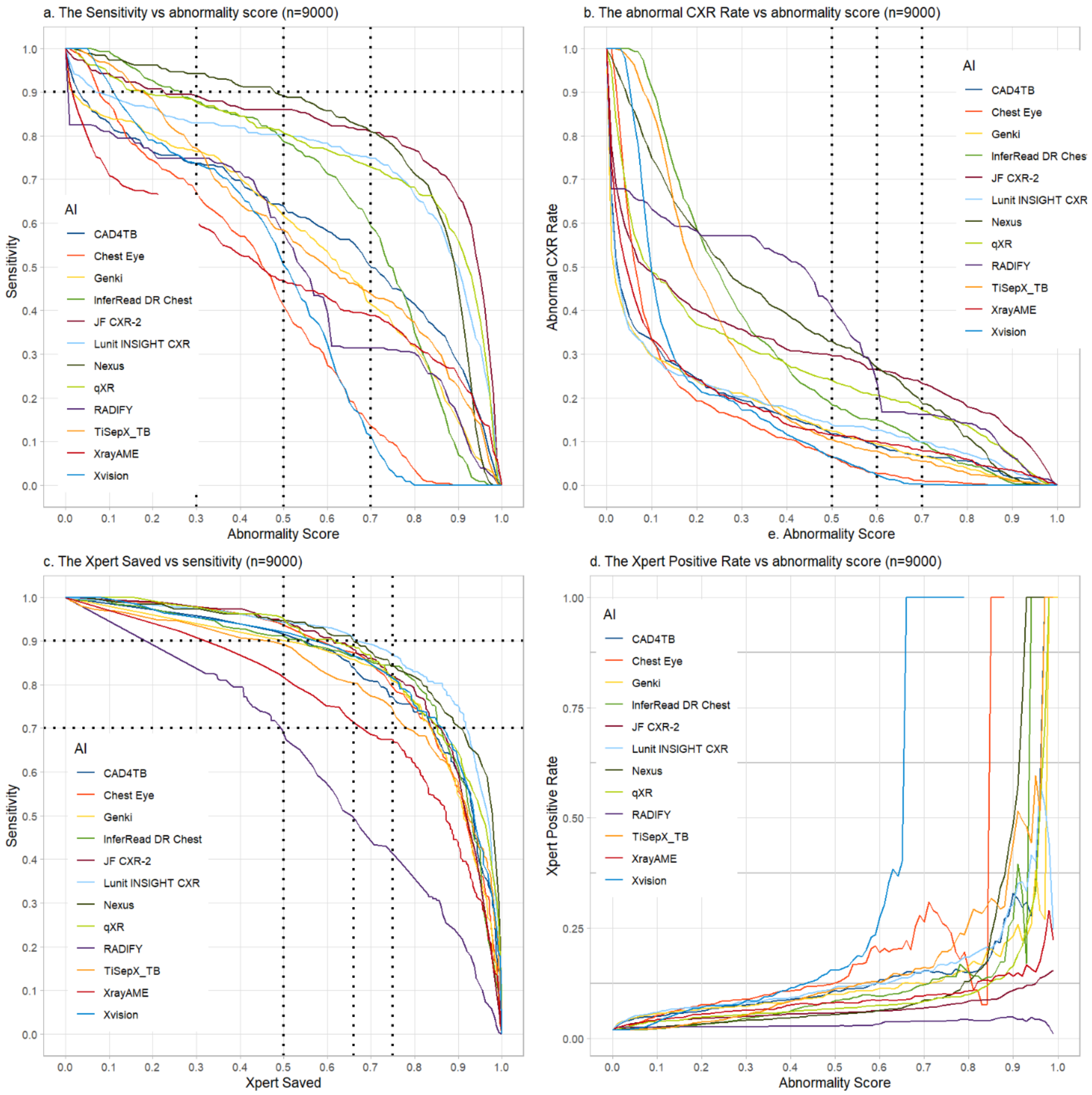

Further analysis:

At the midpoint threshold (0.5), we saw wide variation in the abnormal CXR rate, as well as differences in sensitivity and specificity. For example, XVision and ChestEye flagged less than 10% of CXR for confirmatory testing, while JF CXR-2 resulted in a CXR abnormality rate of around 30% and qXR around 25%. Only when selecting a threshold of around 0.45 was the abnormal CXR rate for RADIFY greater than 50%.

Compared to CXR positivity rate, increasing the threshold did not result in a significant increase in confirmation test positivity; for most CAD products, the highest gains were seen at thresholds of 0.9 or higher. All products except XrayAME and RADIFY would be able to halve the number of individuals with presumptive TB referred to testing while maintaining sensitivity close to 90% and would save two thirds of TB presumptive individuals referred for testing while keeping the sensitivity greater than 80%.

Annex 10. The AUCs and the p-value of different subgroups
Highlight indicates a p-value <0.05

CAD Software	P value	HIV Negative	HIV Positive
ChestEye	0.008	0.892	0.782
CAD4TB	0.012	0.885	0.776
InferRead DR Chest	0.014	0.891	0.783
qXR	0.017	0.914	0.823
Xvision	0.020	0.896	0.799
JF CXR-2	0.038	0.889	0.806
Genki	0.052	0.877	0.796
XrayAME	0.087	0.802	0.718
Nexus	0.118	0.916	0.861
Lunit INSIGHT CXR	0.286	0.914	0.879
RADIFY	0.403	0.641	0.596
TiSepX_TB	0.602	0.834	0.854
		**Asymptomatic**	**Symptomatic**
CAD4TB	0.007	0.799	0.883
Genki	0.012	0.793	0.874
Xvision	0.060	0.820	0.879

CONFIDENTIAL

InferRead DR Chest	0.074	0.816	0.873
qXR	0.075	0.848	0.897
ChestEye	0.080	0.819	0.872
JF CXR-2	0.102	0.835	0.882
XrayAME	0.163	0.730	0.784
TiSepX_TB	0.247	0.837	0.795
RADIFY	0.274	0.601	0.648
Lunit INSIGHT CXR	0.559	0.889	0.904
Nexus	0.987	0.891	0.891
		**Male**	**Female**
ChestEye	0.012	0.892	0.821
XrayAME	0.138	0.801	0.746
qXR	0.267	0.893	0.864
InferRead DR Chest	0.282	0.870	0.837
Nexus	0.313	0.911	0.885
TiSepX_TB	0.369	0.836	0.805
JF CXR-2	0.528	0.874	0.856
Genki	0.555	0.855	0.837
CAD4TB	0.605	0.862	0.847
Xvision	0.625	0.874	0.860
RADIFY	0.775	0.633	0.621
Lunit INSIGHT CXR	0.796	0.907	0.900
		**New cases**	**Previously treated cases**
ChestEye	<0.001	0.890	0.744
JF CXR-2	<0.001	0.894	0.753

CONFIDENTIAL

Genki	0.002	0.878	0.752
InferRead DR Chest	0.003	0.878	0.761
qXR	0.005	0.904	0.804
Xvision	0.009	0.881	0.783
CAD4TB	0.013	0.869	0.775
Nexus	0.049	0.910	0.844
Lunit INSIGHT CXR	0.065	0.916	0.858
XrayAME	0.339	0.758	0.716
RADIFY	0.640	0.618	0.640
TiSepX_TB	0.936	0.819	0.822
JF CXR-2	0.028	0.928	0.863
		**Young (15 to <35 years)**	**Middle age (35 to <55)**
qXR	0.051	0.941	0.890
ChestEye	0.054	0.921	0.861
Lunit INSIGHT CXR	0.071	0.949	0.903
InferRead DR Chest	0.122	0.907	0.855
TiSepX_TB	0.138	0.888	0.834
Xvision	0.150	0.919	0.875
Genki	0.169	0.905	0.861
Nexus	0.257	0.939	0.909
CAD4TB	0.432	0.896	0.871
XrayAME	0.463	0.817	0.786
RADIFY	0.464	0.676	0.639
		**Young (15 to <35 years)**	**Older (55 years +)**

CONFIDENTIAL

qXR	<0.001	0.941	0.785
Nexus	<0.001	0.939	0.813
ChestEye	<0.001	0.921	0.760
JF CXR-2	<0.001	0.928	0.787
Genki	<0.001	0.905	0.750
TiSepX_TB	<0.001	0.888	0.722
Lunit INSIGHT CXR	<0.001	0.949	0.826
Xvision	<0.001	0.919	0.780
CAD4TB	0.001	0.896	0.759
InferRead DR Chest	0.003	0.907	0.786
XrayAME	0.003	0.817	0.668
RADIFY	0.075	0.676	0.580
		**Middle age (35 to <55)**	**Older (55 years +)**
qXR	0.004	0.890	0.785
CAD4TB	0.005	0.871	0.759
Nexus	0.007	0.909	0.813
Genki	0.007	0.861	0.750
ChestEye	0.013	0.861	0.760
TiSepX_TB	0.015	0.834	0.722
XrayAME	0.016	0.786	0.668
Xvision	0.017	0.875	0.780
Lunit INSIGHT CXR	0.035	0.903	0.826
JF CXR-2	0.049	0.863	0.787
InferRead DR Chest	0.098	0.855	0.786
RADIFY	0.267	0.639	0.580

CONFIDENTIAL

		Non-smoker	Smoker
Xvision	0.011	0.825	0.898
Genki	0.015	0.806	0.880
Nexus	0.015	0.868	0.929
ChestEye	0.032	0.819	0.881
qXR	0.034	0.849	0.904
Lunit INSIGHT CXR	0.035	0.874	0.926
CAD4TB	0.040	0.817	0.878
XrayAME	0.041	0.724	0.800
TiSepX_TB	0.042	0.784	0.855
JF CXR-2	0.054	0.831	0.885
InferRead DR Chest	0.086	0.822	0.875
RADIFY	0.835	0.624	0.633

CONFIDENTIAL

Annex 11: Sensitivity and Specificity for each CAD in each subgroup at a threshold of 0.5

CAD4TB (threshold =50)

**Sensitivity**		**Specificity**	
New cases	History of TB	New cases	History of TB
**59.3% (51.4-66.8%)**	**72.5% (62.2-81.4%)**	**94.2% (91.4-96.2%)**	**70.8% (61.1-79.2%)**
HIV-	HIV+	HIV-	HIV+
**65.3% (57.1-72.9%)**	**63.1% (50.2-74.7%)**	**92.9% (89.6-95.4%)**	**81.8% (71.4-89.7%)**
Female	Male	Female	Male
**51.7% (42.3-61.0%)**	**74.3% (66.2-81.3%)**	**93.8% (90.2-96.4%)**	**84.8% (79.8-89.0%)**
Symptomatic	Asymptomatic	Symptomatic	Asymptomatic
**65.5% (55.8-74.3%)**	**62.8% (54.5-70.6%)**	**94.9% (91.8-97.1%)**	**81.9% (76.2-86.7%)**
Smoker	Non-smoker	Smoker	Non-smoker
**74.8% (66.6-81.9%)**	**52.0% (42.8-61.1%)**	**86.0% (80.5-90.4%)**	**91.5% (87.8-94.4%)**

Sensitivity			Specificity		
Young age (15<35 years)	Middle age (35<55 years)	Old age (55 years +)	Young age (15<35 years)	Middle age (35<55 years)	Old age (55 years +)
**70.2 (59.3-79.7%)**	**70.9% (61.1-79.4%)**	**46.5% (34.5-58.7%)**	**86.7% (80-91.8%)**	**72.2% (64.7-79%)**	**87.7% (82.5-91.8%)**

Chest Eye (threshold =0.5)

**Sensitivity**		**Specificity**	
New cases	History of TB	New cases	History of TB
**33.5% (26.4-1.2%)**	**54.9% (44.2-65.4%)**	**97.8% (95.9-99.0%)**	**80.2% (71.3-87.3%)**
HIV-	HIV+	HIV-	HIV+

CONFIDENTIAL

**48.7% (40.4-57.0%)**	**35.4% (23.9- 48.2%)**	**95.3% (92.4-97.3%)**	**93.5% (85.5-97.9%)**
Female	Male	Female	Male
**27.1% (19.3-36.1%)**	**52.9% (44.2- 61.3%)**	**96.5% (93.5-98.4%)**	**91.8% (87.8-94.9%)**
Symptomatic	Asymptomatic	Symptomatic	Asymptomatic
**35.5% (26.6-45.1%)**	**45.3% (37.1- 53.7%)**	**96.6% (93.9-98.4%)**	**91.0% (86.4-94.4%)**
Smoker	Non-smoker	Smoker	Non-smoker
**54.8% (46.0-63.4%)**	**26.0% (18.5- 34.7%)**	**90.8% (86.0-94.4%)**	**96.4% (93.7-98.2%)**

Sensitivity			Specificity		
Young age (15<35 years)	Middle age (35<55 years)	Old age (55 years +)	Young age (15<35 years)	Middle age (35<55 years)	Old age (55 years +)
**41.7% (31.0-52.9%)**	**52.4% (42.4 - 62.4%)**	**23.9% (14.6-35.5%)**	**94.8% (89.6-97.9%)**	**93.9% (88.7-97.2%)**	**94.0% (90.2-96.7%)**

Genki (threshold =0.5)

**Sensitivity**		**Specificity**	
New cases	History of TB	New cases	History of TB
**56.9% (49.0 - 64.5%)**	**70.3% (59.8- 79.5%)**	**93.7% (90.9 - 95.8%)**	**68.9% (59.1 - 77.5%)**
HIV-	HIV+	HIV-	HIV+
**64.7% (56.5 - 72.3%)**	**61.5% (48.6- 73.3%)**	**90.6% (86.9- 93.5%)**	**85.7% (75.9- 92.6%)**
Female	Male	Female	Male
**49.2% (39.8- 58.5%)**	**72.1% (63.9- 79.4%)**	**94.6% (91.1 - 97.0%)**	**82.5% (77.3- 86.9%)**
Symptomatic	Asymptomatic	Symptomatic	Asymptomatic
**64.5% (54.9 - 73.4%)**	**59.5% (51.1- 67.4%)**	**94.3% (91.0- 96.6%)**	**81.0% (75.2- 85.9%)**
Smoker	Non-smoker	Smoker	Non-smoker

CONFIDENTIAL

**74.8% (66.6 - 81.9%)**	**47.2% (38.1 - 56.4%)**	**85.5% (80.0- 90.0%)**	**90.6% (86.7 - 93.6%)**

Sensitivity			Specificity		
Young age (15<35 years)	Middle age (35<55 years)	Old age (55 years +)	Young age (15<35 years)	Middle age (35<55 years)	Old age (55 years +)
**67.9% (56.8 - 77.6%)**	**71.8% (62.1- 80.3%)**	**39.4% (28.0- 51.7%)**	**93.3% (87.7- 96.9%)**	**86.4% (79.8- 91.5%)**	**87.2% (82.3 - 91.2%)**

InferRead (threshold =0.5)

**Sensitivity**		**Specificity**	
New cases	History of TB	New cases	History of TB
**77.2% (70.1-83.4%)**	**81.3% (71.8-88.7%)**	**90.3% (87.0-93.0%)**	**53.8% (43.8-63.5%)**
HIV-	HIV+	HIV-	HIV+
**85.3% (78.6-90.6%)**	**69.2% (56.6-80.1%)**	**85.8% (81.7-89.4%)**	**76.6% (65.6-85.5%)**
Female	Male	Female	Male
**66.9% (57.7-75.3%)**	**88.6% (82.1-93.3%)**	**88.5% (83.9-92.1%)**	**77.0% (71.4-82.0%)**
Symptomatic	Asymptomatic	Symptomatic	Asymptomatic
**74.5% (65.4-82.4%)**	**81.8% (74.6-87.6%)**	**90.9% (87.0-93.9%)**	**71.9% (65.5-77.8%)**
Smoker	Non-smoker	Smoker	Non-smoker
**88.1% (81.5-93.1%)**	**68.3% (59.3-76.4%)**	**78.3% (72.0-83.7%)**	**85.7% (81.2-89.4%)**

Sensitivity			Specificity		
Young age (15<35 years)	Middle age (35<55 years)	Old age (55 years +)	Young age (15<35 years)	Middle age (35<55 years)	Old age (55 years +)

CONFIDENTIAL

82.1% (72.3-89.6%)	82.5% (73.8-89.3%)	69.0% (56.9-79.5%)	88.1% (81.5-93.1%)	79.6% (72.2-85.8%)	81.7% (76.2-86.4%)

JF CXR-2 **(threshold =0.5)**

**Sensitivity**		**Specificity**	
New cases	History of TB	New cases	History of TB
**83.8% (77.4-89.1%)**	**90.1% (82.1-95.4%)**	**81.5% (77.4- 85.1%)**	**32.1% (23.3- 41.8%)**
HIV-	HIV+	HIV-	HIV+
**88.7% (82.5- 93.3%)**	**84.6% (73.5- 92.4%)**	**76.1% (71.2- 80.5%)**	**59.7% (47.9- 70.8%)**
Female	Male	Female	Male
**78.0% (69.4- 85.1%)**	**92.9% (87.3- 96.5%)**	**75.8% (70.1- 80.8%)**	**66.9% (60.8 - 72.6%)**
Symptomatic	Asymptomatic	Symptomatic	Asymptomatic
**80.0% (71.3 - 87.0%)**	**90.5% (84.6 - 94.7%)**	**82.4% (77.6 - 86.6%)**	**56.6% (49.7 - 63.2%)**
Smoker	Non-smoker	Smoker	Non-smoker
**94.1% (88.7 - 97.4%)**	**77.2% (68.8 - 84.3%)**	**68.6% (61.8 - 74.9%)**	**73.0% (67.6 - 77.9%)**

Sensitivity			Specificity		
Young age (15<35 years)	Middle age (35<55 years)	Old age (55 years +)	Young age (15<35 years)	Middle age (35<55 years)	Old age (55 years +)
**89.3% (80.6 - 95.0%)**	**90.3% (82.9 - 95.2%)**	**76.1% (64.5 - 85.4%)**	**85.9% (78.9 - 91.3%)**	**63.9% (55.6-71.7%)**	**67.7% (61.3- 73.6%)**

Lunit **(threshold =0.5)**

**Sensitivity**	**Specificity**

CONFIDENTIAL

New cases	History of TB	New cases	History of TB
**75.4% (68.2 - 81.8%)**	**89.0% (80.7 - 94.6%)**	**93.7% (90.9- 95.8%)**	**64.2% (54.3- 73.2%)**
HIV-	HIV+	HIV-	HIV+
**84.7% (77.9 - 90.0%)**	**76.9% (64.8 - 86.5%)**	**88.8% (84.9 - 91.9%)**	**84.4% (74.4 - 91.7%)**
Female	Male	Female	Male
**72.0% (63.0- 79.9%)**	**87.1% (80.4 - 92.2%)**	**93.5% (89.7 - 96.1%)**	**81.7% (76.4 - 86.2%)**
Symptomatic	Asymptomatic	Symptomatic	Asymptomatic
**78.2% (69.3 - 85.5%)**	**81.8% (74.6 - 87.6%)**	**92.6% (89.0 - 95.3%)**	**81.0% (75.2 - 85.9%)**
Smoker	Non-smoker	Smoker	Non-smoker
**88.1% (81.5 - 93.1%)**	**71.5% (62.7 - 79.3%)**	**82.6% (76.7 - 87.5%)**	**90.9% (87.1 - 93.9%)**

Sensitivity			Specificity		
Young age (15<35 years)	Middle age (35<55 years)	Old age (55 years +)	Young age (15<35 years)	Middle age (35<55 years)	Old age (55 years +)
**91.7% (83.6- 96.6%)**	**83.5% (74.9 - 90.1%)**	**62.0% (49.7 - 73.2%)**	**88.9% (82.3 - 93.6%)**	**84.4% (77.5 - 89.8%)**	**88.9% (84.2 - 92.6%)**

Nexus (threshold =0.5)

**Sensitivity**		**Specificity**	
New cases	History of TB	New cases	History of TB
**88.6% (82.8-93.0%)**	**90.1% (82.1-95.4%)**	**79.1% (74.8-82.9%)**	**26.4% (18.3-35.9%)**
HIV-	HIV+	HIV-	HIV+
**90.7% (84.8-94.8%)**	**89.2% (79.1-95.6%)**	**70.5% (65.3-75.3%)**	**54.5% (42.8-65.9%)**
Female	Male	Female	Male
**82.2% (74.1-88.6%)**	**95.0% (90.0-98.0%)**	**75.8% (70.1-80.8%)**	**60.7% (54.4-66.7%)**

CONFIDENTIAL

Symptomatic	Asymptomatic	Symptomatic	Asymptomatic
**83.6% (75.4-90.0%)**	**93.2% (87.9-96.7%)**	**77.0% (71.8-81.7%)**	**56.6% (49.7-63.2%)**
Smoker	Non-smoker	Smoker	Non-smoker
**94.8% (89.6-97.9%)**	**82.9% (75.1-89.1%)**	**61.4% (54.4-68.0%)**	**72.6% (67.3-77.5%)**

Sensitivity			Specificity		
Young age (15<35 years)	Middle age (35<55 years)	Old age (55 years +)	Young age (15<35 years)	Middle age (35<55 years)	Old age (55 years +)
**91.7% (83.6-96.6%)**	**93.2% (86.5-97.2%)**	**80.3% (69.1-88.8%)**	**82.2% (74.7-88.3%)**	**59.9% (51.5-67.9%)**	**65.5% (59.1-71.6%)**

qXR **(threshold =0.5)**

**Sensitivity**		**Specificity**	
New cases	History of TB	New cases	History of TB
**77.2% (70.1-83.4%)**	**86.8% (78.1-93.0%)**	**87.3% (83.7-90.4%)**	**37.7% (28.5-47.7%)**
HIV-	HIV+	HIV-	HIV+
**84.0% (77.1-89.5%)**	**78.5% (66.5-87.7%)**	**81.4% (76.9-85.4%)**	**67.5% (55.9-77.8%)**
Female	Male	Female	Male
**71.2% (62.1-79.2%)**	**88.6% (82.1-93.3%)**	**82.7% (77.5-87.1%)**	**71.6% (65.7-77.0%)**
Symptomatic	Asymptomatic	Symptomatic	Asymptomatic
**76.4% (67.3-83.9%)**	**83.8% (76.8-89.3%)**	**87.5% (83.2-91.0%)**	**63.3% (56.6-69.7%)**
Smoker	Non-smoker	Smoker	Non-smoker
**89.6% (83.2-94.2%)**	**70.7% (61.9-78.6%)**	**72.5% (65.8-78.4%)**	**80.1% (75.2-84.4%)**

Sensitivity	Specificity

CONFIDENTIAL

Young age (15<35 years)	Middle age (35<55 years)	Old age (55 years +)	Young age (15<35 years)	Middle age (35<55 years)	Old age (55 years +)
**89.3% (80.6-95.0%)**	**84.5% (76.0-90.9%)**	**64.8% (52.5-75.8%)**	**87.4% (80.6-92.5%)**	**72.8% (64.8-79.8%)**	**74.0% (67.9-79.5%)**

RADIFY (threshold =0.5)

**Sensitivity**		**Specificity**	
New cases	History of TB	New cases	History of TB
**54.5% (46.6-62.2%)**	**63.7% (53.0 - 73.6%)**	**60.3% (55.4 - 65.1%)**	**57.5% (47.6 - 67.1%)**
HIV-	HIV+	HIV-	HIV+
**59.3% (51.0 - 67.3%)**	**55.4% (42.5- 67.7%)**	**59.3% (53.9 - 64.6%)**	**61.0% (49.2- 72.0%)**
Female	Male	Female	Male
**54.2% (44.8 - 63.4%)**	**60.7% (52.1 - 68.9%)**	**62.3% (56.1 - 68.2%)**	**57.2% (50.9 - 63.3%)**
Symptomatic	Asymptomatic	Symptomatic	Asymptomatic
**58.2% (48.4- 67.5%)**	**57.4% (49.0- 65.5%)**	**64.9% (59.1- 70.3%)**	**52.9% (46.1 - 59.7%)**
Smoker	Non-smoker	Smoker	Non-smoker
**58.5% (49.7- 66.9%)**	**56.9% (47.7 - 65.8%)**	**58.9% (51.9 - 65.7%)**	**60.3% (54.5 - 65.8%)**

**Sensitivity**			**Specificity**		
Young age (15<35 years)	Middle age (35<55 years)	Old age (55 years +)	Young age (15<35 years)	Middle age (35<55 years)	Old age (55 years +)
**53.6% (42.4- 64.5%)**	**61.2% (51.1- 70.6%)**	**57.7% (45.4 - 69.4%)**	**66.7% (58.0- 74.5%)**	**59.2% (50.8 - 67.2%)**	**56.2% (49.6- 62.6%)**

CONFIDENTIAL

TiSep X-TB **(threshold =0.5)**

**Sensitivity**		**Specificity**	
New cases	History of TB	New cases	History of TB
**59.3% (51.4% - 66.8%)**	**57.1% (46.3% - 67.5%)**	**90.8% (87.5% - 93.4%)**	**89.6% (82.2% - 94.7%)**
HIV-	HIV+	HIV-	HIV+
**62.0% (53.7- 69.8%)**	**58.5% (45.6- 70.6%)**	**90.6% (86.9- 93.5%)**	**92.2% (83.8- 97.1%)**
Female	Male	Female	Male
**55.9% (46.5- 65.1%)**	**60.7% (52.1- 68.9%)**	**89.6% (85.3- 93.0%)**	**91.4% (87.3- 94.6%)**
Symptomatic	Asymptomatic	Symptomatic	Asymptomatic
**53.6% (43.9- 63.2%)**	**62.2% (53.8- 70.0%)**	**91.9% (88.2- 94.7%)**	**88.7% (83.8- 92.5%)**
Smoker	Non-smoker	Smoker	Non-smoker
**64.4% (55.8- 72.5%)**	**52.0% (42.8- 61.1%)**	**91.3% (86.6- 94.8%)**	**90.2% (86.3- 93.3%)**

Sensitivity			Specificity		
Young age (15<35 years)	Middle age (35<55 years)	Old age (55 years +)	Young age (15<35 years)	Middle age (35<55 years)	Old age (55 years +)
**76.2% (65.7- 84.8%)**	**59.2% (49.1- 68.8%)**	**36.6% (25.5- 48.9%)**	**89.6% (83.2- 94.2%)**	**90.5% (84.5- 94.7%)**	**91.1% (86.7- 94.4%)**

XrayAME **(threshold =0.5)**

**Sensitivity**		**Specificity**	
New cases	History of TB	New cases	History of TB
**39.5% (32.1- 47.4%)**	**60.4% (49.6 - 70.5%)**	**93.9% (91.2%- 96.0%)**	**70.8% (61.1 - 79.2%)**

CONFIDENTIAL

HIV-	HIV+	HIV-	HIV+
**53.3% (45.0 - 61.5%)**	**43.1% (30.8- 56.0%)**	**90.9% (87.3 - 93.7%)**	**83.1% (72.9 - 90.7%)**
Female	Male	Female	Male
**29.7% (21.6- 38.8%)**	**61.4% (52.8- 69.5%)**	**96.2% (93.0- 98.1%)**	**82.1% (76.9- 86.6%)**
Symptomatic	Asymptomatic	Symptomatic	Asymptomatic
**44.5% (35.1 - 54.3%)**	**48.6% (40.4 - 57.0%)**	**94.9% (91.8- 97.1%)**	**81.4% (75.7- 86.3%)**
Smoker	Non-smoker	Smoker	Non-smoker
**60.0% (51.2- 68.3%)**	**32.5% (24.4 - 41.6%)**	**84.1% (78.3- 88.8%)**	**92.5% (89.0- 95.2%)**

Sensitivity			Specificity		
Young age (15<35 years)	Middle age (35<55 years)	Old age (55 years +)	Young age (15<35 years)	Middle age (35<55 years)	Old age (55 years +)
**51.2% (40.0- 62.3%)**	**50.5% (40.5- 60.5%)**	**36.6% (25.5- 48.9%)**	**93.3% (87.7- 96.9%)**	**87.1% (80.6- 92.0%)**	**88.1% (83.2- 91.9%)**

XVision (threshold =0.5)

**Sensitivity**		**Specificity**	
New cases	History of TB	New cases	History of TB
**46.1% (38.4- 54.0%)**	**58.2% (47.4- 68.5%)**	**97.3% (95.3- 98.7%)**	**83.0% (74.5- 89.6%)**
HIV-	HIV+	HIV-	HIV+
**54.0% (45.7- 62.2%)**	**49.2% (36.6- 61.9%)**	**95.3% (92.4- 97.3%)**	**92.2% (83.8- 97.1%)**
Female	Male	Female	Male
**38.1% (29.4- 47.5%)**	**60.7% (52.1- 68.9%)**	**97.7% (95.0 - 99.1%)**	**91.1% (86.9- 94.2%)**
Symptomatic	Asymptomatic	Symptomatic	Asymptomatic

CONFIDENTIAL

**52.7% (43.0- 62.3%)**	**48.6% (40.4- 57.0%)**	**98.0% (95.6- 99.3%)**	**89.6% (84.8- 93.3%)**
Smoker	Non-smoker	Smoker	Non-smoker
**62.2% (53.5- 70.4%)**	**37.4% (28.8- 46.6%)**	**92.3% (87.8- 95.5%)**	**95.8% (92.9- 97.7%)**

Sensitivity			Specificity		
Young age<br>(15<35 years)	Middle age<br>(35<55 years)	Old age<br>(55 years +)	Young age<br>(15<35 years)	Middle age<br>(35<55 years)	Old age<br>(55 years +)
**57.1% (45.9- 67.9%)**	**55.3% (45.2- 65.1%)**	**35.2% (24.2- 47.5%)**	**96.3% (91.6- 98.8%)**	**95.2% (90.4- 98.1%)**	**92.8% (88.7- 95.7%)**

CONFIDENTIAL

Annex 12: Different CAD software specificity and threshold at pre-defined sensitivity levels and disaggregated by age groups, HIV status and prior TB history.

Sensitivity		New Case	With TB History	HIV-	HIV+	Young Age	Middle Age	Old Age
						15<35 years	35<55 years	≤55 years
**CAD4TB**								
**90%**	**Threshold**	**2**	**7**	**4**	**2**	**3**	**6**	**1**
	**Specificity**	**55.7% (50.8-60.6%)**	**35.8% (26.8-45.7%)**	**64.6%(59.3-69.7%)**	**36.4% (25.7-48.1%)**	**69.6% (61.1-77.2%)**	**62.6% (54.2-70.4%)**	**31.1% (25.2-37.4%)**
**80%**	**Threshold**	**11**	**26**	**19***	**9**	**27**	**37**	**4**
	**Specificity**	**76.9% (72.5-80.9%)**	**47.2 (37.4-57.1%)**	**79.4% (74.6-83.5%)**	**51.9% (40.3-63.5%)**	**85.2% (78.1-90.7%)**	**80.3% (72.9-86.4%)**	**53.2% (46.6-59.7%)**
**ChestEye**								
**90%**	***Threshold***	*0.07*	*0.11*	*0.13*	*0.07*	*0.13*	*0.07*	*0.06*
	**Specificity**	63.7% (58.9-68.4%)	29.2% (20.8-38.9%)	78.2% (73.4-82.5%)	46.8% (35.3-58.5%)	88.1% (81.5-93.1%)	51.7% (43.3-60.0%)	35.7% (29.6-42.2%)
**80%**	***Threshold***	*0.13*	*0.23*	*0.23*	*0.10*	*0.24*	*0.21*	*0.10*
	**Specificity**	84.2% (80.3-87.6%)	50.0% (40.1-59.9%)	85.8% (81.7-89.4%)	55.8% (44.1-67.2%)	90.4% (84.1-94.8%)	75.5% (67.7-82.2%)	62.1% (55.6-68.4%)
**Genki**								
**90%**	***Threshold***	*0.01*	*0.02*	*0.03*	*0.01*	*0.02*	*0.04*	*0.01*
	**Specificity**	55.7% (50.8-60.6%)	22.6% (15.1-31.8%)	63.7% (58.3-68.8%)	42.9% (31.6-54.6%)	75.6% (67.4-82.5%)	60.5% (52.2-68.5%)	34.5% (28.4-40.9%)
**80%**	***Threshold***	*0.17*	*0.34*	*0.25*	*0.10*	*0.31*	*0.39*	*0.05*
	**Specificity**	85.6% (81.9-88.9%)	52.8% (42.9-62.6%)	83.8% (79.4-87.5%)	61.0% (49.2-72.0%)	89.6% (83.2-94.2%)	81.0% (73.7-87.0%)	56.2% (49.6-62.6%)
**InferRead**								
**90%**	***Threshold***	*0.24*	*0.28*	*0.44*	*0.18*	*0.37*	*0.28*	*0.19*

CONFIDENTIAL

Sensitivity		New Case	With TB History	HIV-	HIV+	Young Age 15<35 years	Middle Age 35<55 years	Old Age ≤55 years
	**Specificity**	58.2% (53.2-63.0%)	26.4% (18.3-35.9%)	81.4% (76.9-85.4%)	27.3% (17.7-38.6%)	81.5% (73.9-87.6%)	57.1% (48.7-65.3%)	32.3% (26.4-38.7%)
**80%**	***Threshold***	*0.46*	*0.52*	*0.59*	*0.39*	*0.58*	*0.54*	*0.32*
	**Specificity**	87.8% (84.3-90.8%)	53.8% (43.8-63.5%)	87.3% (83.3-90.7%)	64.9% (53.2-75.5%)	90.4% (84.1-94.8%)	82.3% (75.2-88.1%)	61.3% (54.7-67.5%)
**JF CXR-2**								
**90%**	***Threshold***	*0.22*	*0.56*	*0.36*	*0.16*	*0.36*	*0.55*	*0.13*
	**Specificity**	72.3% (67.7-76.5%)	33.0% (24.2-42.8%)	73.2% (68.1-77.8%)	42.9% (31.6-54.6%)	83.0% (75.5-88.9%)	67.3% (59.1-74.8%)	43.8% (37.4-50.4%)
**80%**	***Threshold***	*0.60*	*0.85*	*0.85*	*0.58*	*0.85*	*0.83*	*0.37*
	**Specificity**	84.9% (81.1-88.2%)	55.7% (45.7-65.3%)	87.0% (83.0-90.4%)	63.6% (51.9-74.3%)	91.1% (85.0-95.3%)	78.9% (71.4-85.2%)	64.7% (58.2-70.8%)
**Lunit**								
**90%**	***Threshold***	*0.06*	*0.44*	*0.11*	*0.08*	*0.72*	*0.14*	*0.04*
	**Specificity**	77.6% (73.3-81.6%)	59.4% (49.5-68.9%)	77.3% (72.5-81.6%)	57.1% (45.4-68.4%)	91.1% (85.0-95.3%)	69.4% (61.3-76.7%)	52.8% (46.2-59.3%)
**80%**	***Threshold***	*0.27*	*0.74*	*0.74*	*0.41*	*0.82*	*0.71*	*0.12*
	**Specificity**	87.6% (84.0-90.6%)	83.0% (74.5-89.6%)	92.6% (89.3-95.2%)	74.0% (62.8-83.4%)	93.3% (87.7-96.9%)	89.8% (83.7-94.2%)	70.6% (64.4 - 76.4%)
**Nexus**								
**90%**	***Threshold***	*0.47*	*0.54*	*0.61*	*0.54*	*0.70*	*0.62*	*0.34*
	Specificity	77.4% (73.0-81.3%)	29.2% (20.8-38.9%)	79.1% (74.3-83.3%)	55.8% (44.1-67.2%)	89.6% (83.2-94.2%)	66.0% (57.7-73.6%)	47.7% (41.1-54.3%)

CONFIDENTIAL

Sensitivity		New Case	With TB History	HIV-	HIV+	Young Age	Middle Age	Old Age
						15<35 years	35<55 years	≤55 years
80%	***Threshold***	0.66	0.79	0.77	0.69	0.83	0.77	0.50
	Specificity	87.8% (84.3-90.8%)	75.5% (66.2-83.3%)	89.1% (85.3-92.2%)	70.1% (58.6-80.0%)	94.8% (89.6-97.9%)	82.3% (75.2-88.1%)	65.5% (59.1-71.6%)
				**qXR**				
**90%**	***Threshold***	*0.14*	*0.34*	*0.30*	*0.10*	*0.34*	*0.38*	*0.13*
	**Specificity**	66.2% (61.4-70.7%)	33.0% (24.2-42.8%)	74.6% (69.6-79.2%)	44.2% (32.8-55.9%)	84.4% (77.2-90.1%)	69.4% (61.3-76.7%)	41.3% (34.9-47.9%)
**80%**	***Threshold***	*0.42*	*0.73*	*0.65*	*0.49*	*0.84*	*0.65*	*0.29*
	**Specificity**	84.7% (80.0-88.0%)	61.3% (51.4-70.6%)	85.0% (80.7-88.6%)	66.2% (54.6-76.6%)	95.6% (90.6-98.4%)	81.0% (73.7-87.0%)	60.0% (53.4-66.3%)
				**RADIFY**				
**90%**	***Threshold***	*0.06*	*0.07*	*0.07*	*NA*	*0.15*	*0.07*	*NA*
	**Specificity**	34.8% (30.2-39.6%)	33.0% (24.2-42.8%)	35.7% (30.6-41.0%)	-	45.2% (36.6-54.0%)	35.4% (27.7-43.7%)	-
**80%**	***Threshold***	*0.07*	*0.2*	*0.17*	*0.1**	*0.33*	*0.15*	*NA*
	**Specificity**	35.5% (30.9-40.4%)	37.7% (28.5-47.7%)	41.0% (35.7-46.4%)	39.0% (28.0-50.8%)	47.4% (38.8-56.2%)	41.5% (33.4-49.9%)	-
				**TiSep-X TB**				
**90%**	***Threshold***	*0.16*	*0.2*	*0.19*	*0.20*	*0.26*	*0.20*	*0.11*
	**Specificity**	39.9% (35.1-44.8%)	52.8% (42.9-62.6%)	49.6% (44.1-55.0%)	55.8% (44.1-67.2%)	57.8% (49.0-66.2%	51.0% (42.7-59.3%)	21.3% (16.2-27.1%)
**80%**	***Threshold***	*0.27*	*0.27*	*0.31*	*0.30*	*0.47*	*0.30*	*0.18*
	**Specificity**	67.4% (62.6-71.9%)	69.8% (60.1-78.3%)	75.2% (70.3-79.7%)	72.7% (61.4-82.3%)	86.7% (79.7-91.9%)	68.7% (60.5-76.1%)	55.3% (48.7-61.8%)
				**Xray AME**				

CONFIDENTIAL

Sensitivity		New Case	With TB History	HIV-	HIV+	Young Age 15<35 years	Middle Age 35<55 years	Old Age ≤55 years
**90%**	***Threshold***	*0.01*	*0.06*	*0.02*	*0.03*	*0.03*	*0.04*	*0.01*
	**Specificity**	29.7% (25.3-34.4%)	32.1% (23.3-41.8%)	36.9% (31.7-42.3%)	36.4% (25.7-48.1%)	41.5% (33.1-50.3%)	44.9% (36.7-53.3%)	*32.3% (26.4-38.7%)*
**80%**	***Threshold***	*0.03*	*0.21*	*0.08*	*0.09*	*0.08*	*0.09*	*0.02**
	**Specificity**	50.6% (45.7-55.5%)	47.2% (37.4-57.1%)	65.5% (60.2-70.5%)	48.1% (36.5-59.7%)	67.4% (58.8-75.2%)	57.8% (49.4-65.9%)	*42.1% (35.7-48.7%)*
**Xvision**								
**90%**	***Threshold***	*0.11*	*0.16*	*0.14*	*0.09*	*0.12*	*0.12*	*0.08*
	**Specificity**	67.4% (62.6-71.9%)	36.8% (27.6-46.7%)	73.5% (68.4-78.1%)	33.8% (23.4-45.4%)	75.6% (67.4-82.5%)	58.5% (50.1-66.6%)	31.1% (25.2-37.4%)
**80%**	***Threshold***	*0.15*	*0.34*	*0.32*	*0.14*	*0.34*	*0.27*	*0.14**
	**Specificity**	81.3% (77.2-84.9%)	57.5% (47.6-67.1%)	86.4% (82.3-89.9%)	61.0% (49.2-72.0%)	93.3% (87.7-96.9%)	76.2% (68.5-82.8%)	65.5% (59.1-71.6%)

Annex 13: Sensitivity and specificity of each CAD across the entire threshold abnormality score range in age (young age [15<35 years], middle age [35<55 years], and old age [55+ years]), HIV, and prior TB history subgroups.

A) CAD4TB (*The abnormality score of CAD4TB is converted to between 0 and 1, instead of 0 and 100.)*

CONFIDENTIAL

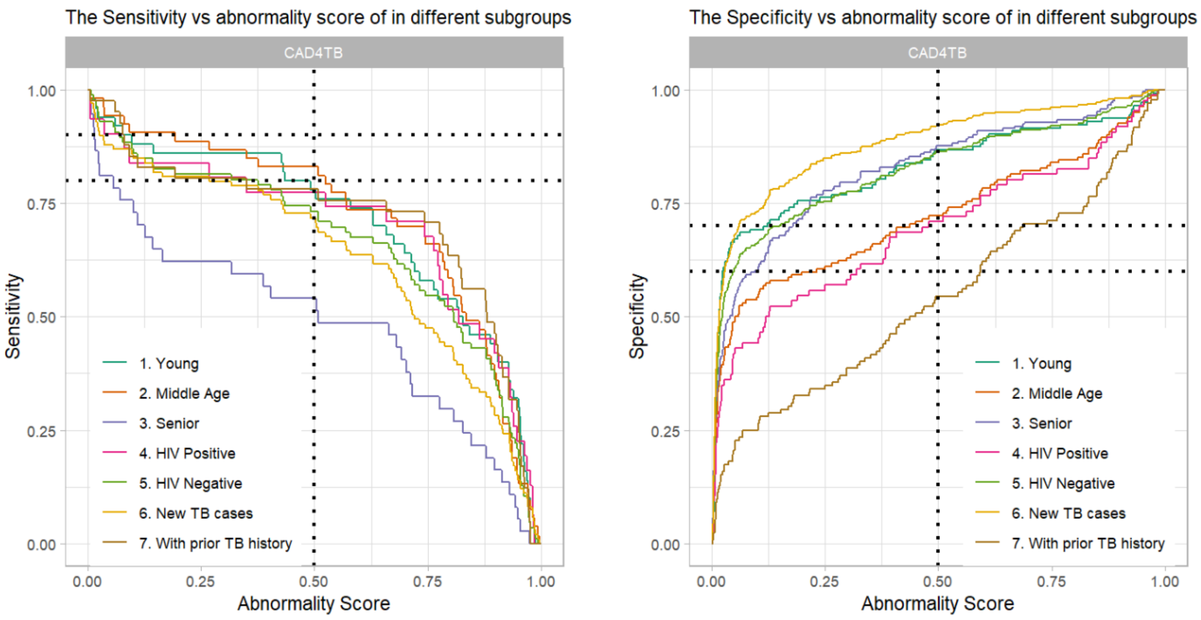

B) Chest Eye

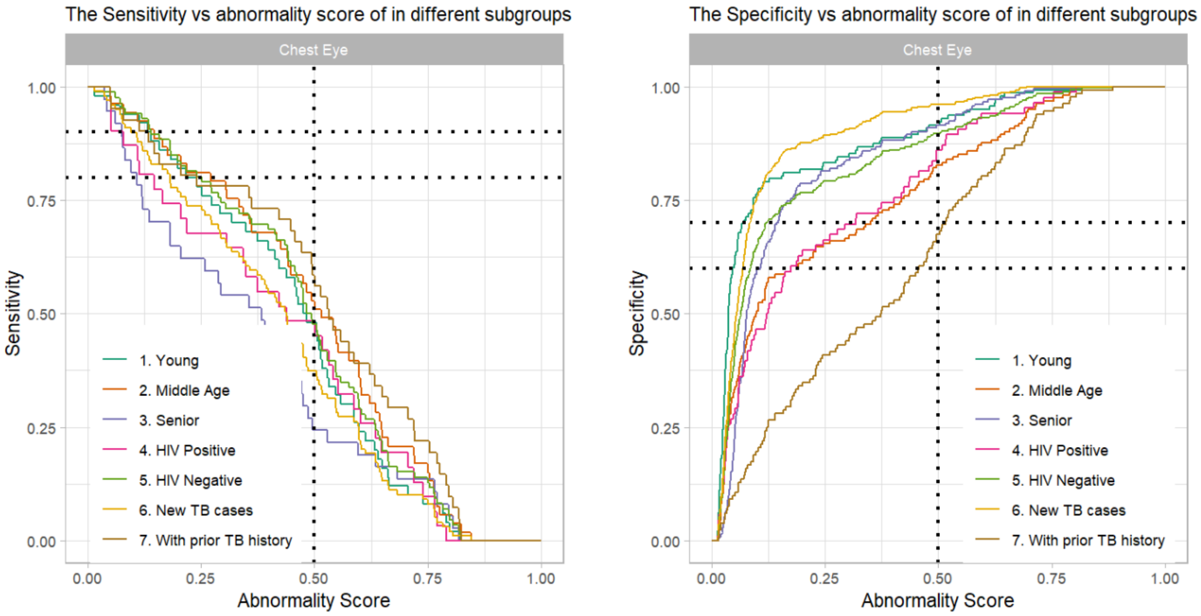

C) Genki

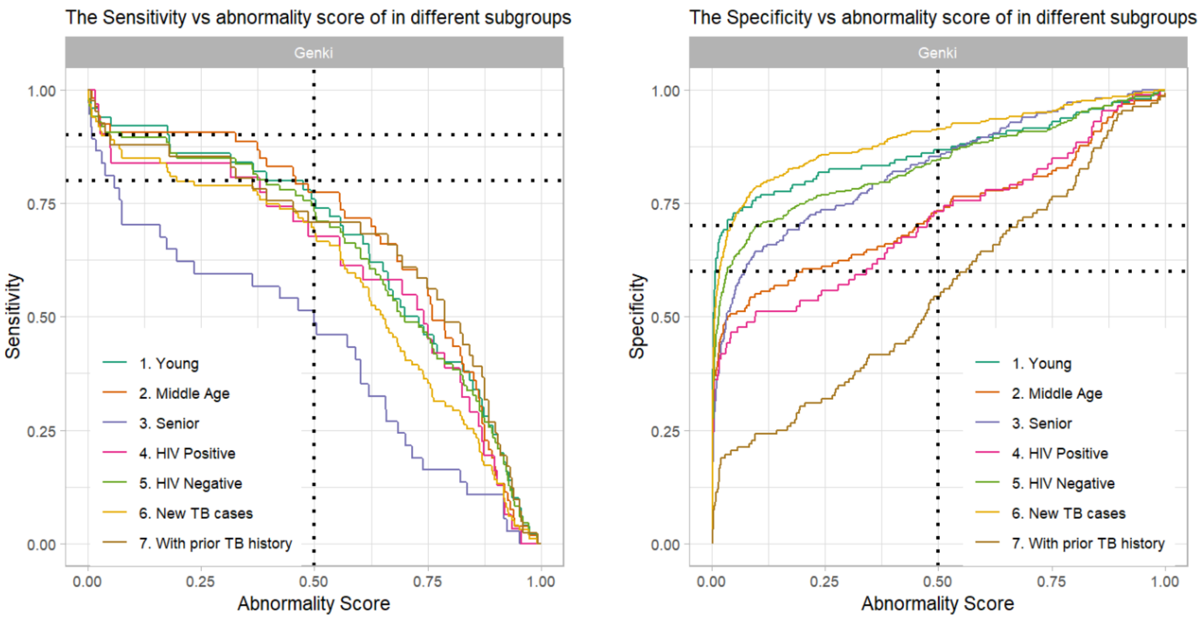

D) InferRead DR Chest

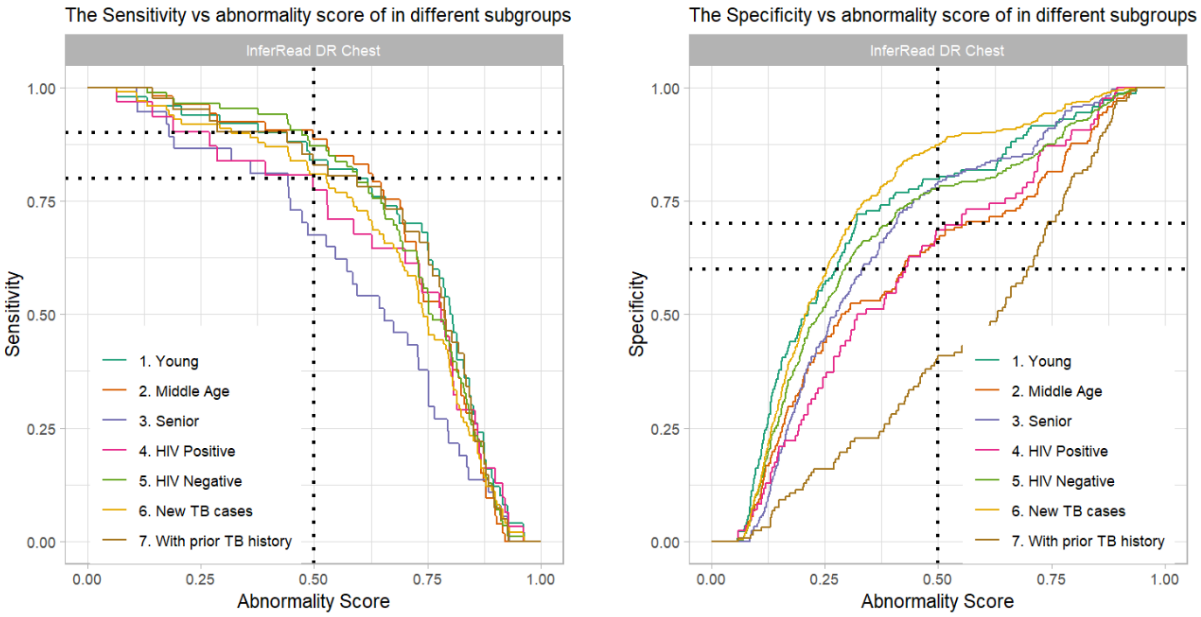

E) JF CXR-2

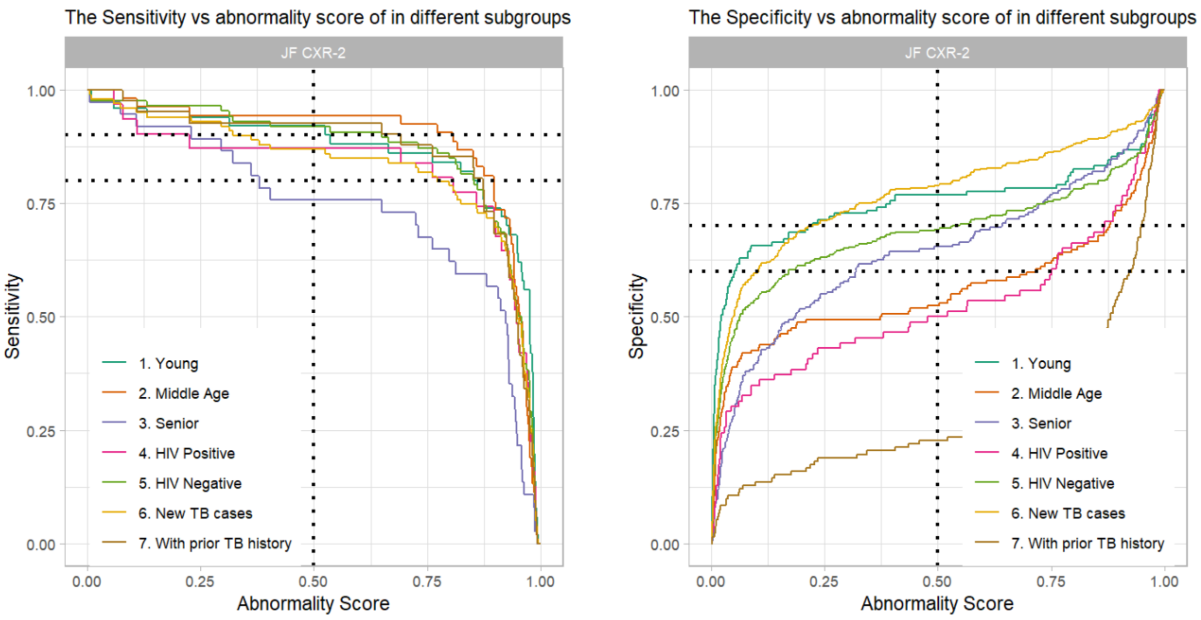

F) Lunit INSIGHT CXR

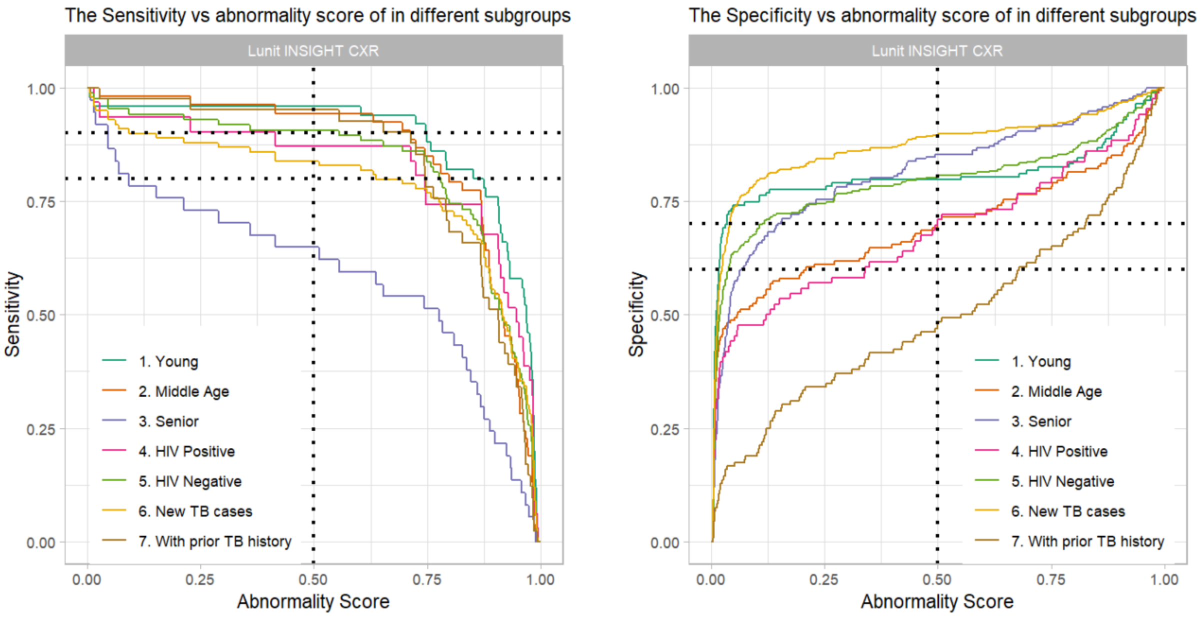

G) Nexus

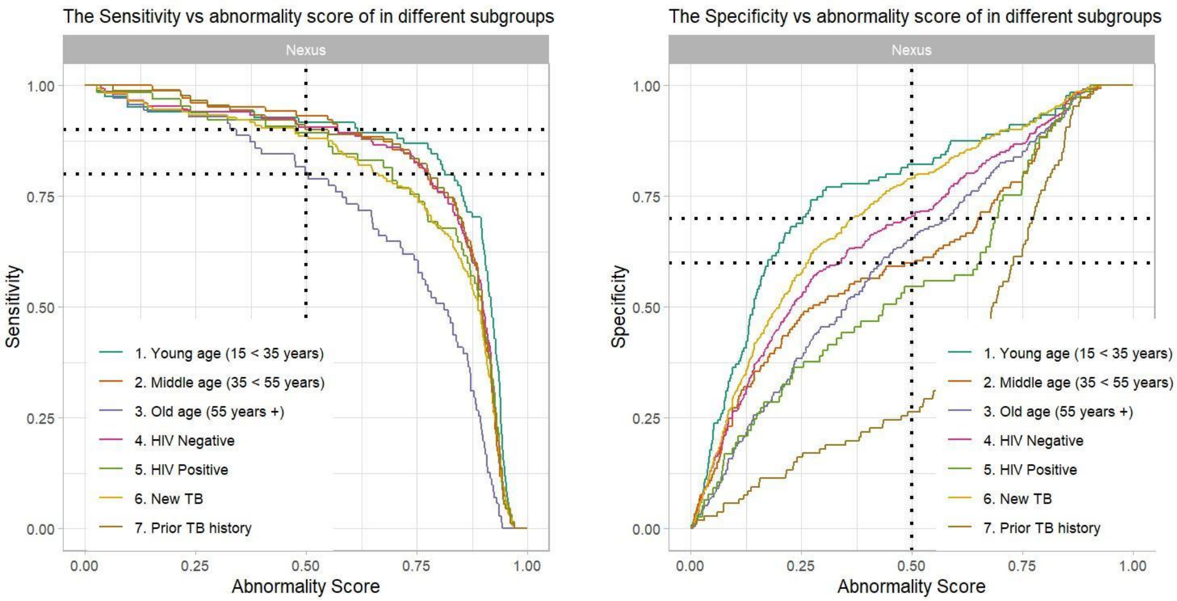
The Sensitivity vs abnormality score of in different subgroups
Nexus
Sensitivity
Abnormality Score
1. Young age (15 < 35 years)
2. Middle age (35 < 55 years)
3. Old age (55 years +)
4. HIV Negative
5. HIV Positive
6. New TB
7. Prior TB history
The Specificity vs abnormality score of in different subgroups
Nexus
Specificity
Abnormality Score
1. Young age (15 < 35 years)
2. Middle age (35 < 55 years)
3. Old age (55 years +)
4. HIV Negative
5. HIV Positive
6. New TB
7. Prior TB history

H) qXR

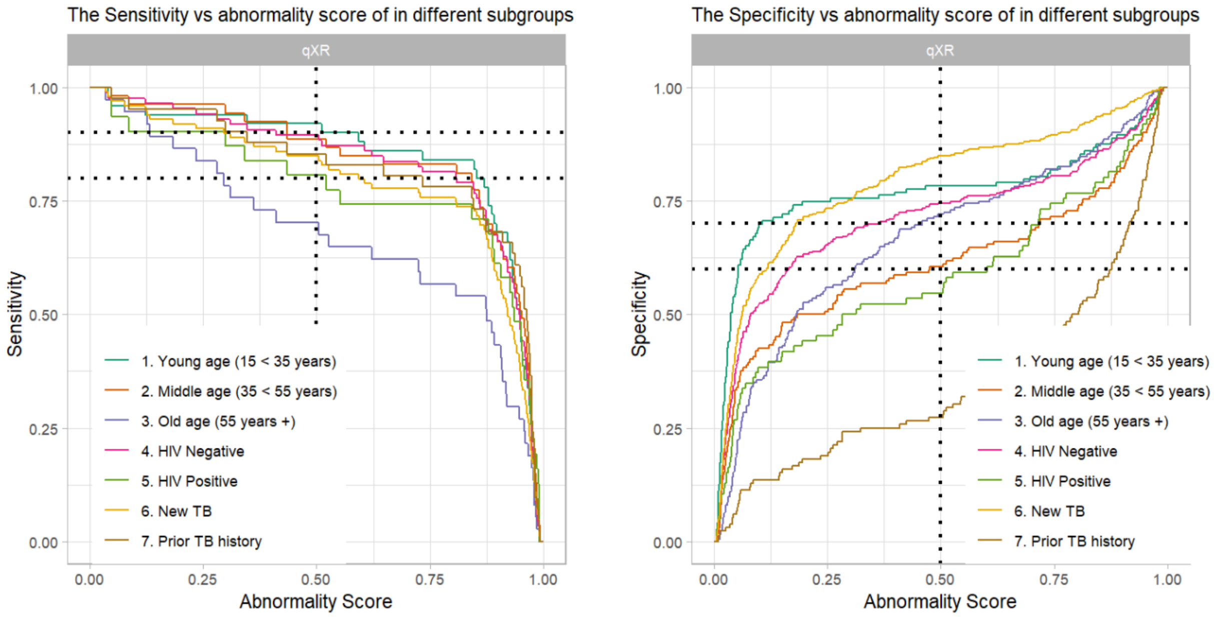
The Sensitivity vs abnormality score of in different subgroups
qXR
Sensitivity
Abnormality Score
1. Young age (15 < 35 years)
2. Middle age (35 < 55 years)
3. Old age (55 years +)
4. HIV Negative
5. HIV Positive
6. New TB
7. Prior TB history
The Specificity vs abnormality score of in different subgroups
qXR
Specificity
Abnormality Score
1. Young age (15 < 35 years)
2. Middle age (35 < 55 years)
3. Old age (55 years +)
4. HIV Negative
5. HIV Positive
6. New TB
7. Prior TB history

I) RADIFY

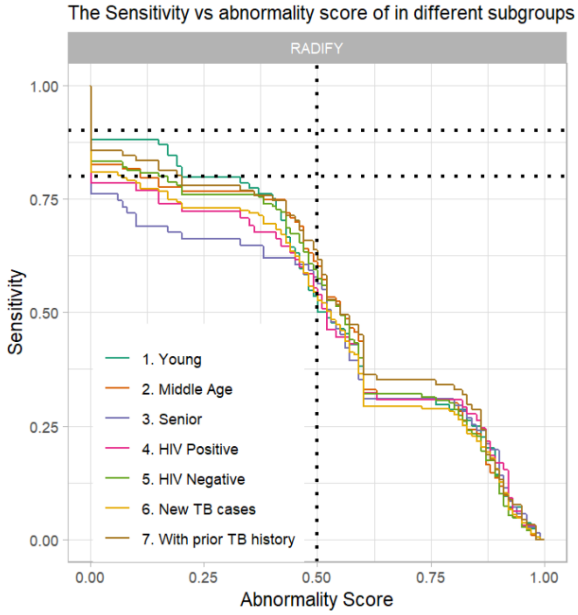

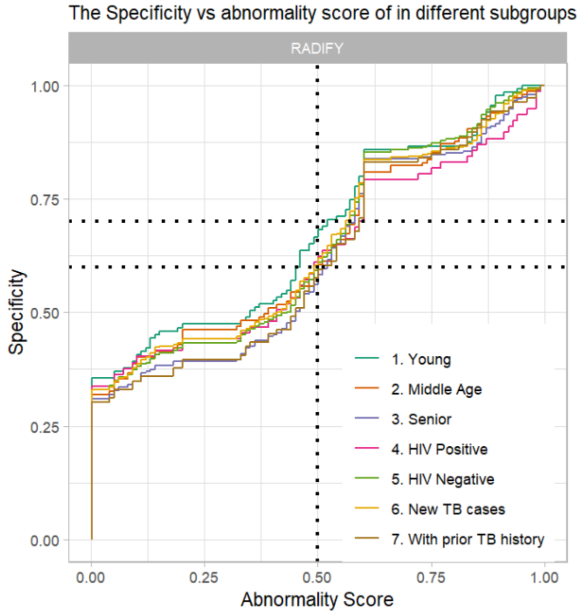

J) TiSepX_TB

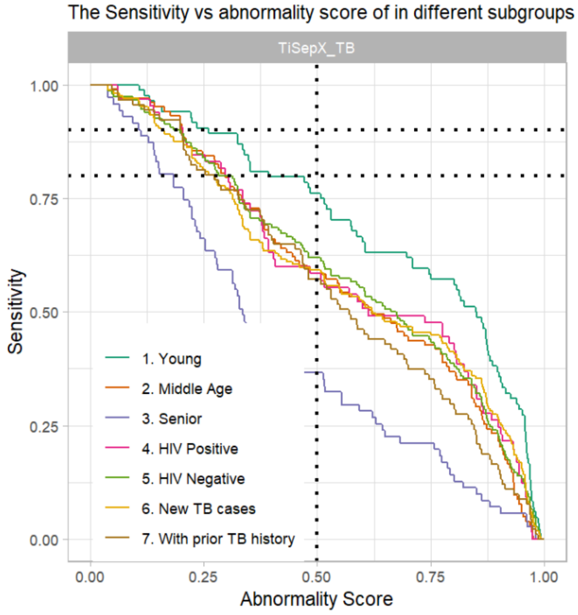

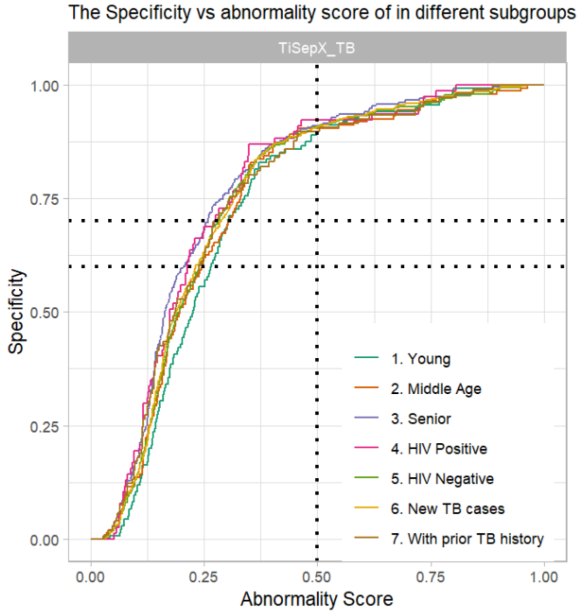

K) XrayAME

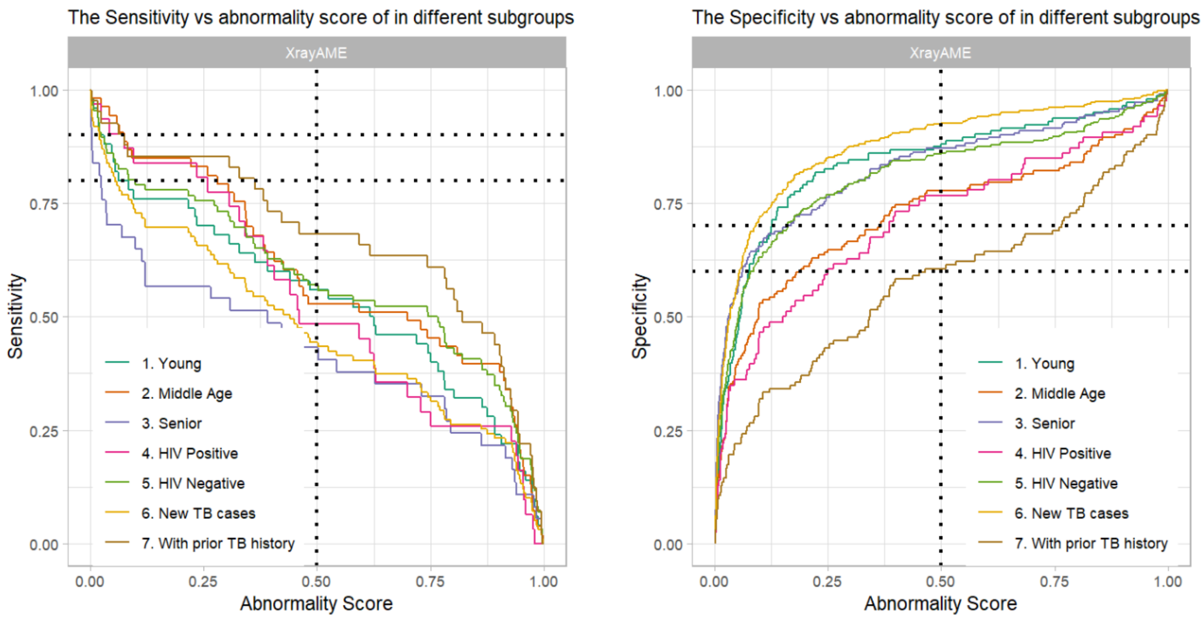
The Sensitivity vs abnormality score of in different subgroups
XrayAME
Sensitivity
Abnormality Score
1.00
0.75
0.50
0.25
0.00
1. Young
2. Middle Age
3. Senior
4. HIV Positive
5. HIV Negative
6. New TB cases
7. With prior TB history
The Specificity vs abnormality score of in different subgroups
XrayAME
Specificity
Abnormality Score

L) XVision

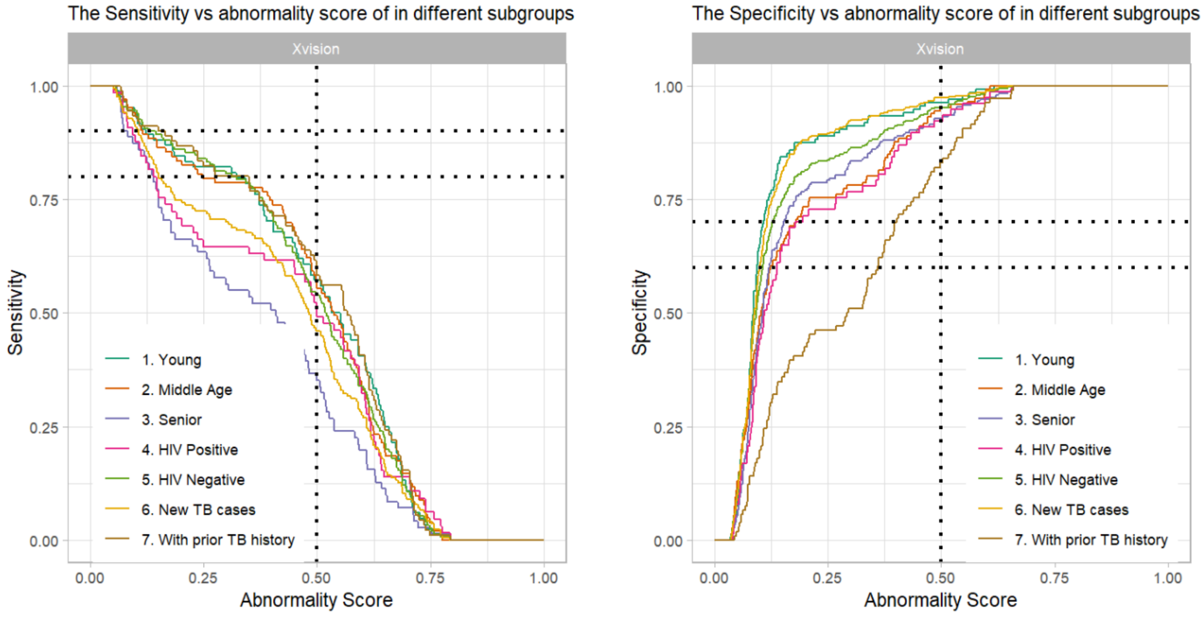
The Sensitivity vs abnormality score of in different subgroups
Xvision
Sensitivity
Abnormality Score
1.00
0.75
0.50
0.25
0.00
1. Young
2. Middle Age
3. Senior
4. HIV Positive
5. HIV Negative
6. New TB cases
7. With prior TB history
The Specificity vs abnormality score of in different subgroups
Xvision
Specificity
Abnormality Score
